# De novo monoallelic Reelin missense variants cause dominant neuronal migration disorders via a dominant-negative mechanism

**DOI:** 10.1172/JCI153097

**Published:** 2024-07-09

**Authors:** Martina Riva, Sofia Ferreira, Kotaro Hayashi, Yoann Saillour, Vera P. Medvedeva, Takao Honda, Kanehiro Hayashi, Claire Altersitz, Shahad Albadri, Marion Rosello, Julie Dang, Malo Serafini, Frédéric Causeret, Olivia J. Henry, Charles-Joris Roux, Céline Bellesme, Elena Freri, Dragana Josifova, Elena Parrini, Renzo Guerrini, Filippo Del Bene, Kazunori Nakajima, Nadia Bahi-Buisson, Alessandra Pierani

**Affiliations:** 1 Université Paris Cité, Institute of Psychiatry and Neuroscience of Paris, INSERM U1266, and; 2 Université Paris Cité, Imagine Institute, Team Genetics and Development of the Cerebral Cortex, Paris, France.; 3 Department of Anatomy, Keio University School of Medicine, Tokyo, Japan.; 4 Sorbonne Université, INSERM U968, CNRS UMR 7210, Institut de la Vision, Paris, France.; 5 Department of Molecular Medicine and Surgery, Karolinska Institute, Stockholm, Sweden.; 6 Pediatric Radiology, Necker Enfants Malades University Hospital, Université de Paris, Paris, France.; 7 Pediatric Neurology, Bicêtre University Hospital, Université Paris Saclay, Kremlin-Bicêtre, France.; 8 Dipartimento di Neuroscienze Pediatriche Fondazione Istituto Neurologico “C. Besta,” Milan, Italy.; 9 Department of Clinical Genetics, Guy’s and St Thomas’ Hospital NHS Trust, London, United Kingdom.; 10 Neuroscience Department, Meyer Children’s Hospital IRCCS, Florence, Italy.; 11 University of Florence, Florence, Italy.; 12 Institut des Sciences Biologiques, Centre National de la Recherche Scientifique (CNRS), Paris, France.; 13 GHU Paris Psychiatrie et Neurosciences, Hôpital Sainte Anne, Paris, France.

**Keywords:** Development, Neuroscience, Cell migration/adhesion, Genetic diseases, Neurodevelopment

## Abstract

Reelin (RELN) is a secreted glycoprotein essential for cerebral cortex development. In humans, recessive *RELN* variants cause cortical and cerebellar malformations, while heterozygous variants were associated with epilepsy, autism, and mild cortical abnormalities. However, the functional effects of *RELN* variants remain unknown. We identified inherited and de novo *RELN* missense variants in heterozygous patients with neuronal migration disorders (NMDs) as diverse as pachygyria and polymicrogyria. We investigated in culture and in the developing mouse cerebral cortex how different variants impacted RELN function. Polymicrogyria-associated variants behaved as gain-of-function, showing an enhanced ability to induce neuronal aggregation, while those linked to pachygyria behaved as loss-of-function, leading to defective neuronal aggregation/migration. The pachygyria-associated de novo heterozygous *RELN* variants acted as dominant-negative by preventing WT RELN secretion in culture, animal models, and patients, thereby causing dominant NMDs. We demonstrated how mutant RELN proteins in vitro and in vivo predict cortical malformation phenotypes, providing valuable insights into the pathogenesis of such disorders.

## Abstract 2

Altered RELN function assessed by in vitro and in vivo assays sheds light on patients’ phenotypes of cortical malformations.

## Graphical abstract

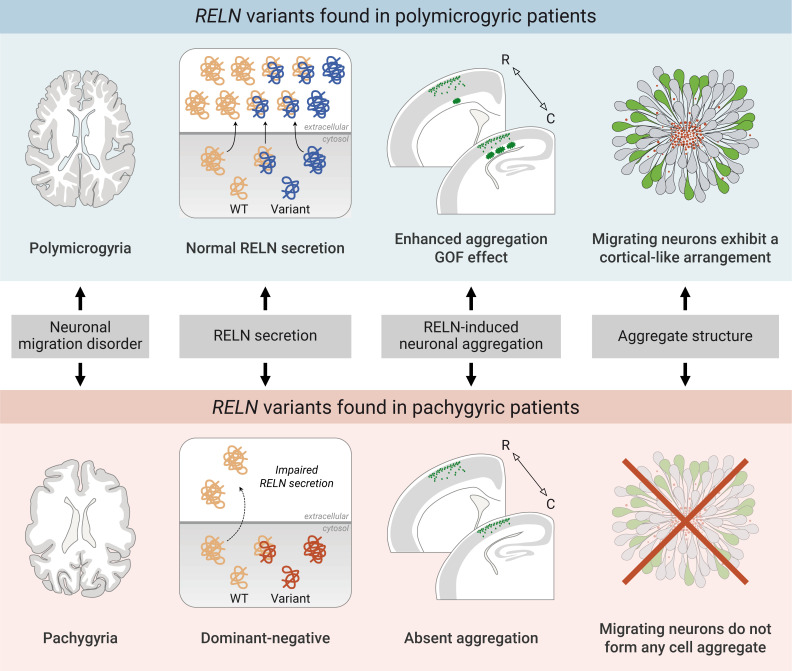

## Introduction

The neocortex is composed of 6 layers that are built during embryonic development through highly orchestrated processes of successive generation of cohorts of glutamatergic neurons in the proliferative zones and their radial migration to form distinct layers (1). The inside-out sequence in the formation of these layers, whereby later-born neurons bypass earlier-born ones to position more superficially, is a unique characteristic of the mammalian neocortex (2). This process relies on the first generated neurons, Cajal-Retzius (CR) cells, which from the cortical surface orchestrate the radial migration, through the secretion of the Reelin (RELN) protein (3, 4). RELN is a large secreted glycoprotein, which is cleaved in the extracellular environment at 2 main specific sites, between repeats 2–3 (N-terminal site) and repeats 6–7 (C-terminal site) (5–8), by cleaving enzymes such as matrix metalloproteinases (6, 9–11). Studies on RELN proteolysis have identified 3 key domains. The N-terminal (N-t) domain is necessary for multimerization (12, 13), while the central region (R3–6) binds to the RELN receptors apolipoprotein E receptor 2 (ApoER2) and very low-density lipoprotein receptor (VLDLR) (5, 14–16). The C-terminus (C-t) contains a small carboxy-terminal region (CTR) and is required for downstream signaling activation (17, 18), but its role in secretion is not fully elucidated yet (18, 19). The full-length protein is generally more efficient in activating the transduction cascade, probably because of the N-t region that promotes homodimerization through disulfide linkage and the CTR that mediates proper folding (12, 13, 18, 20). Although RELN has been studied for almost three decades, its functions are still unclear. On one hand, it is proposed that it acts as an attractant cue (21), and on the other hand it is thought to serve as a “detach and go” signal instructing migrating neurons close to the marginal zone (MZ) to disengage from the radial glia and switch from a locomotion mode of migration to terminal translocation (5, 22–27). RELN has been initially studied via the characterization of the *reeler* (*rl/rl*) homozygous mouse mutant (4, 28), which shows a profound disorganization of cortical lamination, largely due to impaired migration of pyramidal neurons (3, 29). In contrast, heterozygous *reeler* (*rl/+*) mice (haploinsufficient for RELN) show no defects in cortical layering but exhibit a spectrum of cognitive and behavioral abnormalities (30, 31), which emphasizes the relevance of RELN expression levels in higher brain functions.

In humans, recessive *RELN* variants in the homozygous or compound heterozygous state are associated with different patterns of lissencephaly (LIS) with cerebellar hypoplasia (LCH), a severely disabling developmental disorder (32–39), often linked with epilepsy. Fifteen pathogenic or likely pathogenic *RELN* variants in 12 families with this condition have been identified to date, including null alleles and splice-site and missense variants. In addition, one single patient with polymicrogyria, microcephaly, and epilepsy was described with 2 missense variants (40). Several heterozygous *RELN* variants were identified as risk factors for multiple neuropsychiatric and neurodegenerative disorders, such as schizophrenia, bipolar disorders, autism spectrum disorders (ASD), and Alzheimer’s disease (41–43) in the absence of cortical malformations. Moreover, heterozygous *RELN* variants account for 17.5% of familial cases of autosomal dominant lateral temporal lobe epilepsy (ADLTE) with relatively low penetrance (44, 45). These are mainly missense variants, which alter structurally important amino acids predicted to perturb protein folding (44, 45), but they do not lead to brain malformations. Only four ADLTE-causing missense *RELN* variants (46) and one de novo missense variant identified in an ASD patient (47) were functionally characterized in vitro showing reduced secretion of mutated RELN. Recently, monoallelic *RELN* variants, including splice-site and missense variants were reported in 8 families with frontotemporal- or temporal-predominant LIS but with normal cerebellum, (37, 48). However, it is unknown whether the phenotypes arise from gain of function (GOF) or loss of function (LOF) and, importantly, which specific subfunction of RELN may be affected in order to cause such a high variety of pathologies.

Here we report 6 patients with inherited and de novo heterozygous missense *RELN* variants associated with a spectrum of malformations of cortical development (MCDs), namely polymicrogyria (excessive number of abnormally small gyri) or pachygyria (simplified cortical gyral pattern with shallow sulci and broad gyri) (49) without cerebellar hypoplasia. We functionally characterized each variant through a set of in vitro and in vivo assays to assess the secretion of the mutated proteins and their capacity to cause aggregates/rosettes and regulate neuronal migration upon their ectopic expression in the embryonic mouse cerebral cortex. We assessed their pathogenicity, demonstrating that all variants interfere with at least one of the studied processes, and characterizing to what extent that interference correlates with the pathological phenotype. We also provide what we believe to be the first evidence that monoallelic de novo *RELN* variants found in pachygyria patients can cause autosomal dominant neuronal migration disorders (NMDs) by behaving as dominant-negative forms that impair WT RELN secretion in vitro, in animal models, and in patients. Our findings indicate that defects of RELN secretion and function contribute to NMDs, shedding light on the involvement of RELN in the etiology of MCDs.

## Results

### Cortical malformations in patients carrying RELN variants.

**Figure 1:**
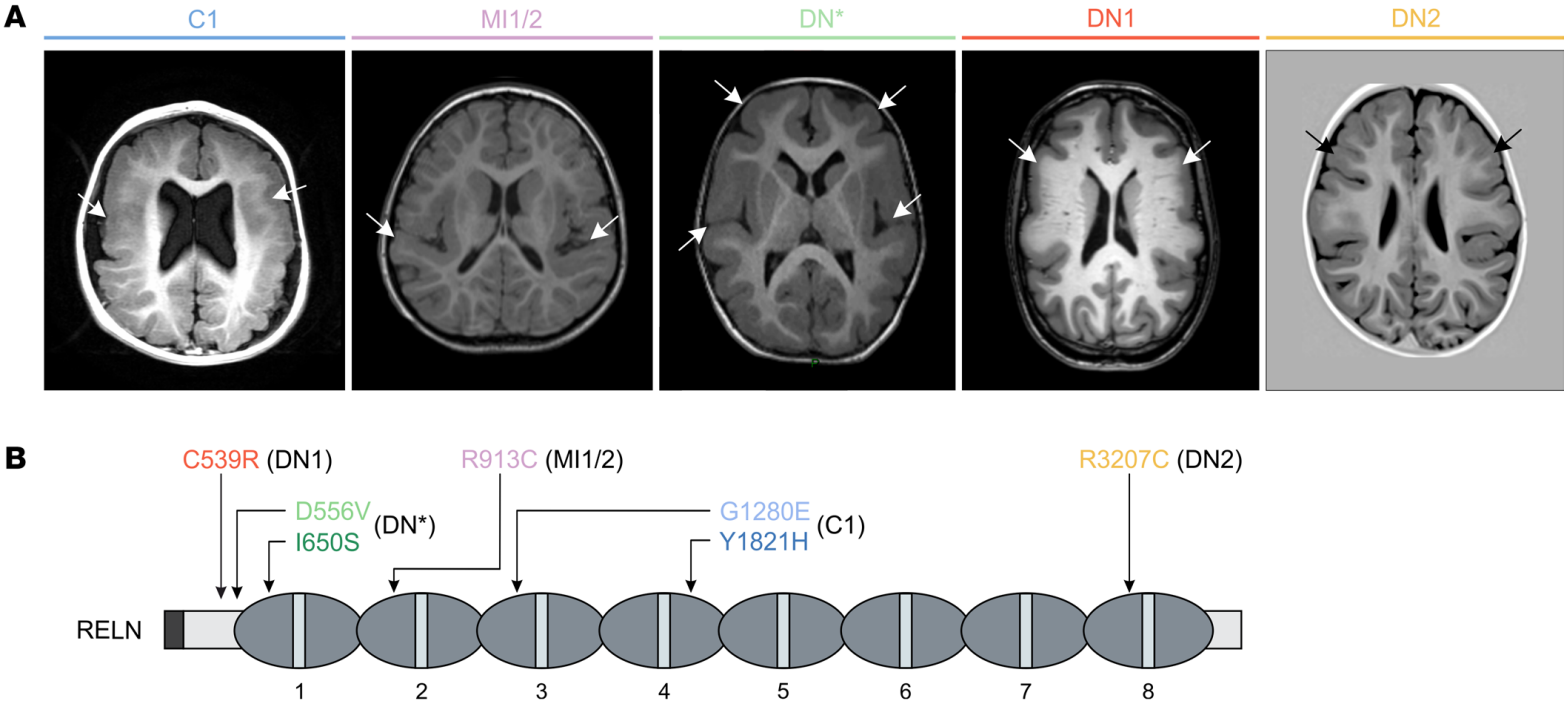
Cortical malformations in heterozygous patients associated with *RELN* missense variants. (**A**) Brain MRI from patients with heterozygous *RELN* variants. C1 exhibits bilateral fronto-parietal polymicrogyria with nodular heterotopia, MI1/2 bilateral perisylvian polymicrogyria, DN* frontal-predominant bilateral pachygyria, and DN1 and DN2 frontotemporal-predominant bilateral pachygyria. Representative axial T1 section of the cortical malformations (white arrows). (**B**) Primary structure of the RELN protein showing 8 Reelin repeats (1–8 ovals). Arrows indicate the position of missense variants; each color corresponds to a patient (C1 blue, MI1/2 pink, DN* green, DN1 orange, and DN2 yellow).

Seven missense *RELN* (NM_005045.4) variants were identified in 6 children with cortical malformations without cerebellar abnormalities (Figure 1, Table 1, and Supplemental Figure 1; supplemental material available online with this article; https://doi.org/10.1172/JCI153097DS1). One child (C1) carries 2 variants, and the other 5 have monoallelic variants: 2 brothers (MI1 and MI2) bearing the same maternally inherited variant, 1 child (DN*) with a paternally inherited plus a de novo variant on the same allele, and, lastly, 2 unrelated children (DN1 and DN2) with de novo variants. Affected children were diagnosed at 1–8 years of age with hypotonia and cognitive developmental delays. The first patient, C1, exhibited bilateral fronto-temporo-parietal polymicrogyria and periventricular nodular heterotopia at brain magnetic resonance imaging (MRI) (Figure 1A). Next-generation sequencing (NGS) analysis of a dedicated panel of genes associated with MCDs revealed 2 missense *RELN* variants, c.5461T>C (p.Tyr1821His) in Reelin repeat 4 (RR4) and c.3839G>A (p.Gly1280Glu) in RR3, denoted as Y1821H and G1280E, respectively (Figure 1B and Table 1). The G1280E substitution was maternally inherited whereas Y1821H was de novo, but given the unavailability of DNA samples from the trio during this study, the presence of the 2 variants on the same allele (as in patient DN*) or on different alleles cannot be assessed. Patients MI1 and MI2, two brothers (hereafter referred to as MI1/2), exhibited MRI imaging consistent with bilateral perisylvian polymicrogyria (Figure 1A). In these 2 patients, an NGS panel for genes associated with MCDs and intellectual disability revealed the c.2737C>T (p.Arg913Cys) missense substitution (R913C) in the RR2 of the *RELN* gene (Figure 1B and Table 1), which they both inherited from their apparently healthy, but unexamined, mother. No other variants of significance were identified by whole-exome sequencing in these brothers. The fourth patient (DN*), exhibiting bilateral pachygyria, which is part of the LIS spectrum, primarily manifested in the frontal regions (Figure 1A), underwent NGS analysis of MCD genes. It revealed the c.1949T>G/c.1667A>T (p.Ile650Ser/p.Asp556Val) missense *RELN* variants (I650S/D556V) with the I650S localized in RR1 and the D556V in the N-t domain (Figure 1B and Table 1). Parental analysis revealed that both variants are in a *cis* configuration on the same paternal allele (see Supplemental Methods). The last 2 patients, hereafter DN1 and DN2, as reported previously (37), presented at the brain MRI bilateral pachygyria with simplified gyral pattern, notably frontotemporal-predominant in the case of DN1 and frontal-predominant for DN2, and becoming less severe posteriorly (Figure 1A). NGS analysis of a panel for MCD genes identified in patient DN1 a de novo c.1615T>C (p.Cys539Arg) variant (C539R) in the N-terminal of the *RELN* gene and a de novo c.9619C>T (p.Arg3207Cys) in DN2 (R3207C) located in RR8 (Figure 1B and Table 1). Most of the *RELN* variants were predicted to be damaging by 2 Web-based programs (PolyPhen-2 [http://genetics.bwh.harvard.edu/pph2/] and Combined Annotation Dependent Depletion [CADD] [https://cadd.bihealth.org/] scores), except the inherited I650S. The majority were also absent from a public reference population database (Genome Aggregation Database [gnomAD] v3.1.2 Non-neuro), whereas the G1280E was present with a 1.2% frequency and the R913C with very low frequency (Table 1). According to the American College of Medical Genetics and Genomics/Association for Molecular Pathology (ACMG/AMP) 2015 guidelines (50), all de novo variants were assessed as likely pathogenic (PS2+PM1+PM2+PP3), the G1280E as benign (PM1+BS1+BS2+BP6), and the R913C (PM1+PM2+PP3) and I650S (PM1+PM2) as variants of uncertain significance (Table 1). All patients had normal comparative genomic hybridization array. With the exception of MI1/2, all patients were born from non-consanguineous healthy parents. Among all patients, only MI1 had epilepsy. All family pedigrees are shown in Supplemental Figure 1.

These results suggest that heterozygous *RELN* variants are associated with a variety of cortical malformations, as diverse as pachygyria, a generalized transmantle migration abnormality, and polymicrogyria, which is still classified as a post-migrational disorder (51), in the absence of cerebellar hypoplasia, previously thought to be the hallmark of RELN-dependent autosomal recessive LIS.

### RELN missense variants reduce its secretion.

**Figure 2:**
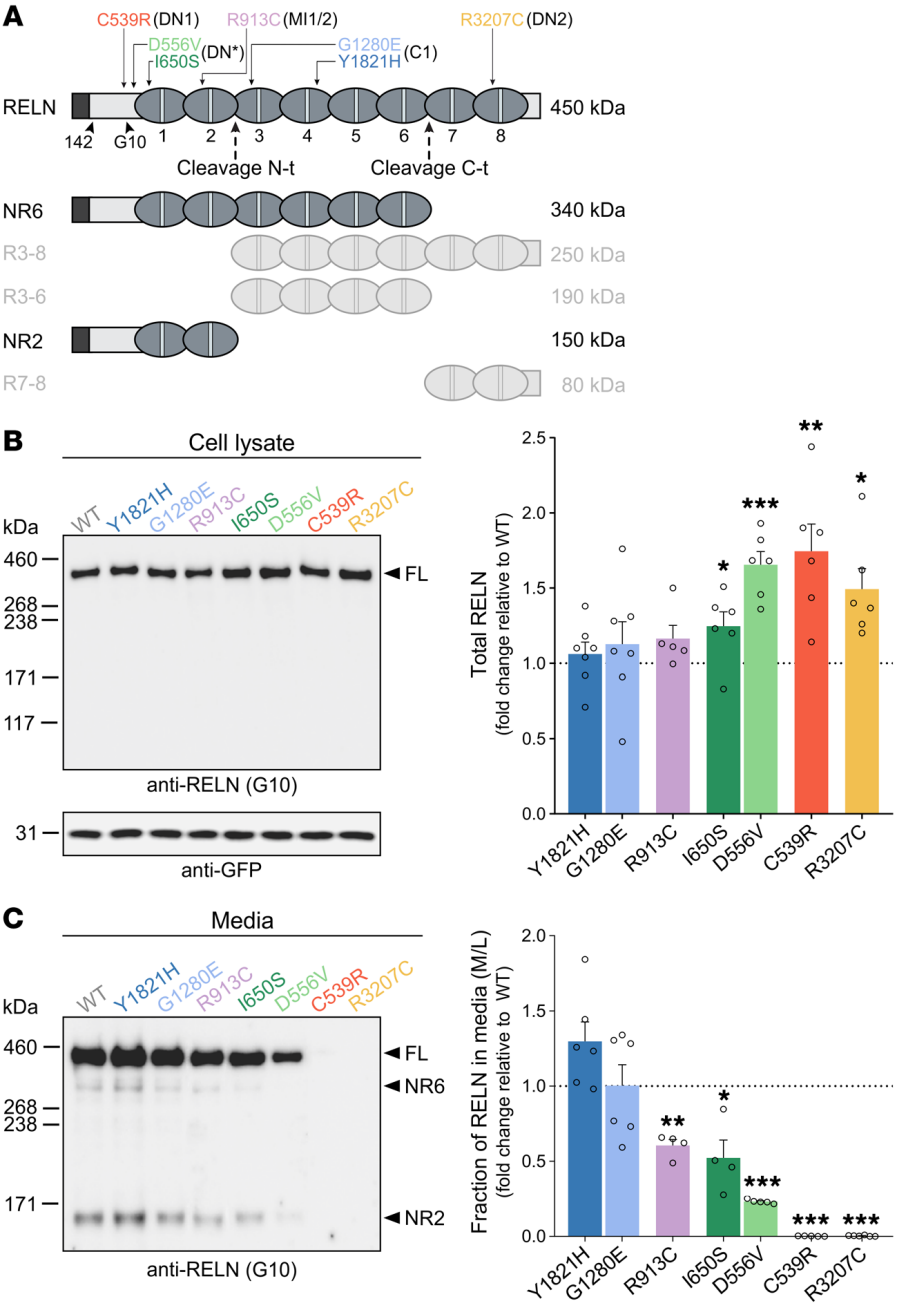
Missense variants alter RELN secretion in vitro. (**A**) Schematic of the full-length (FL) RELN protein (450 kDa), its N-t and C-t cleavage sites (dashed arrows), and its 5 cleaved products (NR6, R3-8, R3-6, NR2, R7-8). The binding regions of the 142 and G10 antibodies and the position of *RELN* variants in the patient color coding are indicated with arrowheads and arrows, respectively. (**B** and **C**) Immunoblots (left) and densitometric analysis (right) of HEK293T cell lysates (**B**) and media (**C**) transfected with either WT-RELN or RELN variants, probed with anti-RELN G10 or anti-GFP antibodies. RELN signal normalized to GFP in lysates (*n* = 5–7 independent transfections) and expressed as the media-to-lysate (M/L) ratio in the media (*n* = 4–6 independent transfections). Data are mean ± SEM; 2-tailed 1-sample *t* test, *P < 0.05, **P < 0.01, ***P < 0.001. kDa, protein standard sizes.

We first investigated whether the missense variants identified in the 6 patients with MCDs could affect RELN expression and/or secretion. We introduced each of the 7 missense variants into the mouse *RELN* sequence (affected residues are conserved but shifted +1 amino acid compared with human; see Supplemental Methods). IRES-eGFP–expressing plasmids carrying the mouse WT-RELN or the different variants were transfected into HEK293T cells, which lack endogenous RELN. RELN levels in both cell lysates and media were compared by immunoblotting using G10 (Figure 2) and 12/14 (Supplemental Figure 2) antibodies, recognizing epitopes in the N-t and the C-t region, respectively (52). GFP-transfected cells showed no signal in either cell lysates or media (data not shown). Upon WT-RELN transfection, a single full-length (FL) 450 kDa band was observed in the cell fraction (Figure 2B), whereas the FL 450 kDa; the 2 complementary fragments resulting from the N-t cleavage, NR2 (150 kDa) and R3-8 (250 kDa); and those resulting from the C-t cleavage, NR6 (340 kDa) and R7-8 (80 kDa), were visible in the secreted fraction (Figure 2, A and C, and Supplemental Figure 2), indicating that WT-RELN is efficiently secreted and processed as expected (53). In cell lysates, significantly increased levels of FL RELN (450 kDa) were observed for I650S-, D556V-, C539R-, and R3207C-transfected cells compared with WT (Figure 2B). In contrast, we observed a 40% decrease of RELN in the media of R913C-transfected cells, and a 48% and 78% reduction in the media of I650S- and D556V-transfected cells, respectively (Figure 2C). A stronger effect was observed for the C539R and R3207C variants, for which both FL and all RELN proteolytic fragments were undetectable in the culture media (Figure 2C). Similar changes in secreted RELN caused by the different variants were detected using the 12/14 antibodies, which recognize the C-t region of the protein (Supplemental Figure 2B).

Taken together, these observations indicate that the de novo variants in the patients with pachygyria and the inherited variants in patients MI1/2 and DN* cause, respectively, strong and mild deficiency in RELN secretion. The significant accumulation of intracellular RELN detected for the I650S, D556V, C539R, and R3207C variants is consistent with their pronounced deficit in RELN secretion.

### RELN variants affect neuronal aggregation along the rostro-caudal axis of the developing cerebral cortex.

**Figure 3:**
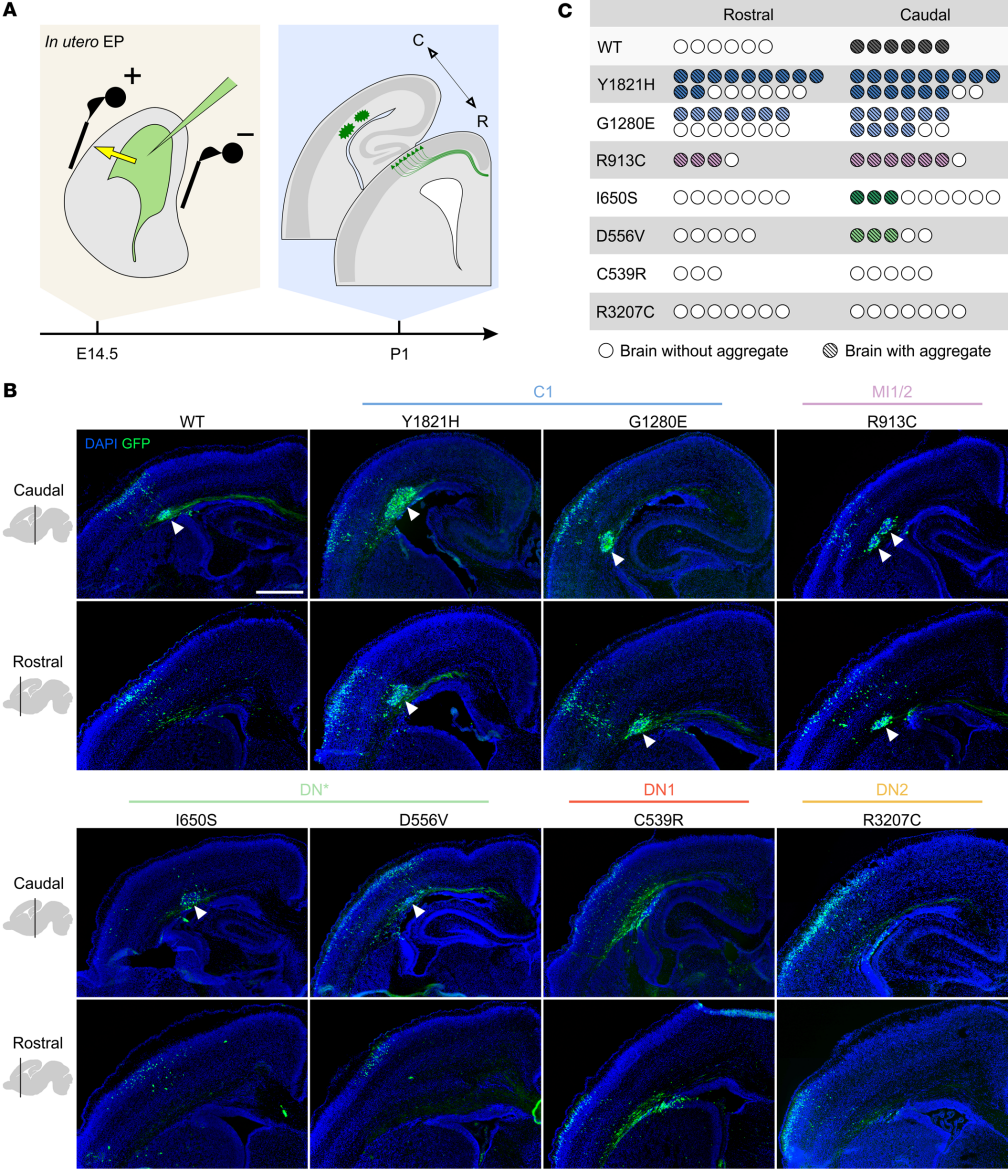
*RELN* variants affect the capacity to form aggregates along the rostro-caudal axis in the embryonic mouse cortex. (**A**) Schematic representation of in utero electroporation (IUE) at E14.5 and collection at P1. (**B**) Wide-field immunofluorescence images of GFP^+^ (green) aggregates (white arrowheads), with DAPI counterstaining (blue), at 2 rostro-caudal levels (bregma 0.86 and –1.58) of P1 mouse brains upon IUE of WT-RELN and patients’ variants, Y1821H, G1280E, R913C, I650S, D556V, C539R, and R3207C. Scale bar: 500 μm. (**C**) Quantification of aggregate formation at rostral and caudal levels for all electroporated constructs (*n* = 3–17 electroporated brains per construct).

RELN is important to regulate neuronal migration and positioning of migrating neurons (3, 4). To test whether *RELN* variants affect its activity in vivo compared with their WT counterpart, we took advantage of a functional assay developed by Kubo et al. (54). Ectopic RELN expression in the developing cortex of mouse embryos drives the radial migration of glutamatergic neurons to form cell aggregates organized around a RELN-rich center, mimicking its production by CR cells in the MZ. We electroporated IRES-eGFP–expressing plasmids carrying WT-RELN or the different variants in the embryonic mouse cortex at E14.5 and collected the brains at postnatal day 1 (P1) (Figure 3A). As previously shown (54), we confirmed that WT-RELN is capable of causing the formation of aggregates (Figure 3B). In addition, we found that these were not forming randomly along the rostro-caudal axis but were forming exclusively in intermediate and caudal regions along the rostro-caudal axis at hippocampal levels (*n* = 6 WT) (Figure 3C). Different effects were obtained when RELN variants were electroporated. I650S and D556V, identified in patient DN* with pachygyria, behaved like the WT with GFP^+^ aggregates forming caudally, although with a lower frequency (I650S, 3 of 9, and D556V, 3 of 5 brains with aggregates), while the Y1821H, G1280E, and R913C variants associated with polymicrogyria promoted the formation of aggregates at both caudal and rostral levels (Figure 3, B and C). Interestingly, the C539R and R3207C variants, found in patients DN1 and DN2, failed to form cell aggregates, consistent with their severely impaired secretion (Figure 2B). All aggregates formed in the intermediate zone (IZ) just below the cortical plate (CP) labeled by TBR1, a marker of deep-layer neurons at this age (Supplemental Figure 3).

Overall, these results allowed us to conclude that (a) aggregates are mostly obtained in posterior regions, indicating that different areas of the developing cortex are not equally responsive to ectopic RELN; (b) variants from patients C1, MI1/2, and DN* lead to the formation of aggregates in the posterior cortex, indicating that they retain some of the activity of the WT protein; (c) polymicrogyria-associated variants from patients C1 and MI1/2 appear to gain the capacity to induce aggregate formation at rostral levels (>50% of brains) and thus represent a GOF in this assay; and (d) the 2 de novo variants from patients DN1 and DN2 behave as complete LOF as shown by the absence of aggregate formation.

### Pachygyria-associated RELN missense variants fail to properly form well-organized rosettes.

**Figure 4:**
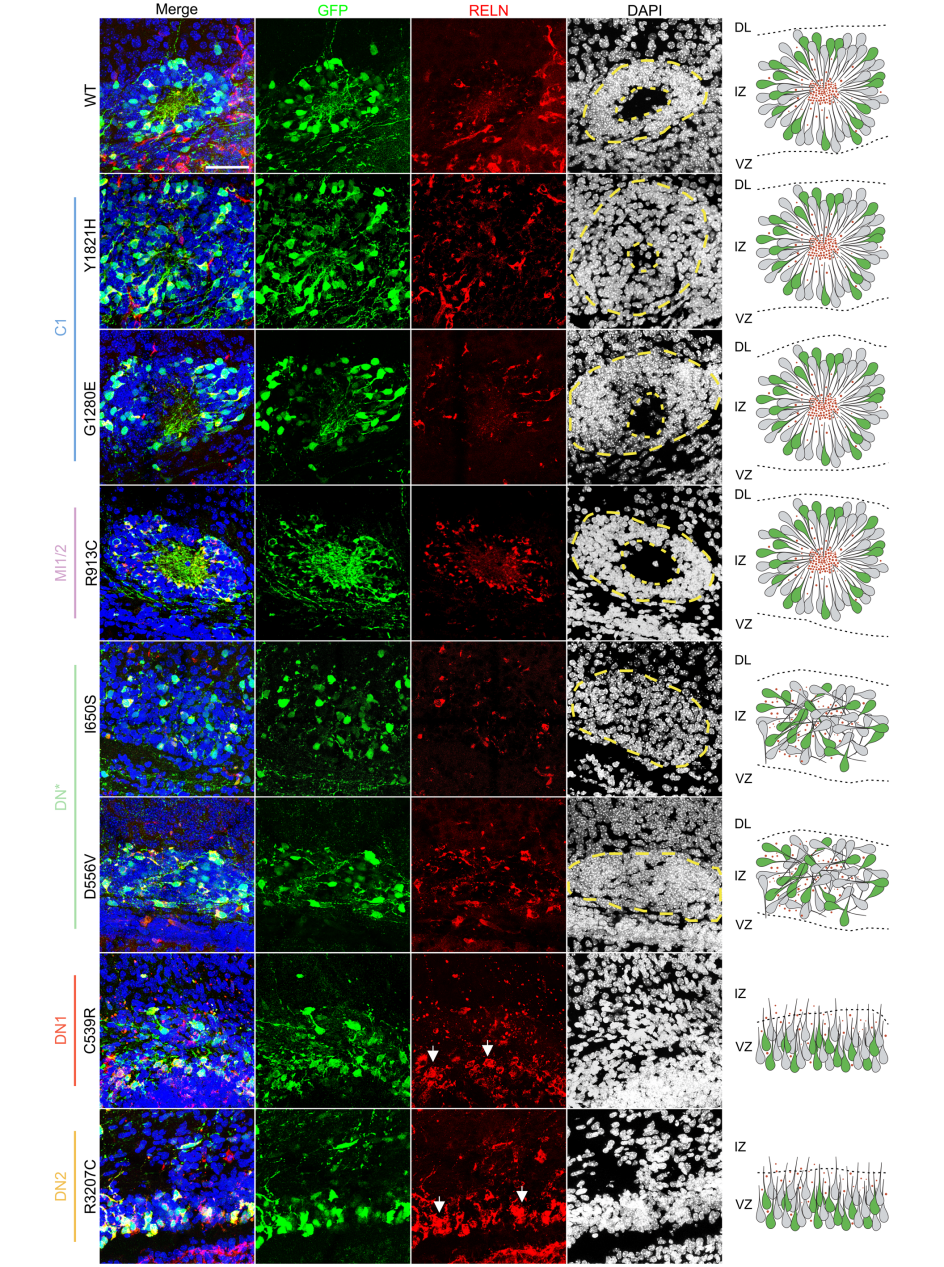
Pachygyria-associated variants fail to generate well-structured rosettes. Immunofluorescence images of aggregates stained with GFP (green) and RELN (red) antibodies and DAPI (blue) for nuclei. Aggregates with electroporated GFP^+^ cells projecting their processes toward a central region that is cell body–poor and RELN-rich are considered properly formed rosettes. Aggregates lacking a central cell body–sparse region with the processes of GFP^+^ cells not projecting radially toward it are simply classified as aggregates. VZ, ventricular zone; IZ, intermediate zone; DL, deeper layers. White arrows indicate GFP^+^ cells with increased RELN signal. Dashed yellow lines outline the rosette/aggregate margins. Scale bar: 50 μm.

 As previously detailed (54), upon WT-RELN electroporation into the developing mouse neocortex at E14.5, spheroid structures are observed at P1.5. These structures, which will be referred to as rosettes, feature electroporated cells radially projecting their processes toward a cell body–poor central region accumulating the RELN protein (Figure 4 and Supplemental Figure 4), analogously to the MZ of the developing cortex. Later-born neurons migrate through early-born neurons to reach the most internal part of this structure, recapitulating, even if ectopically, the inside-out development of the neocortex (54). About 44% of the WT aggregates (12/27) quantified along the rostro-caudal axis of the electroporated brains displayed a rosette structure (Table 2). We thus investigated whether the different missense variants driving the formation of aggregates could effectively generate well-structured rosettes. Our analysis focused on comparing the caudal aggregates obtained with the variants versus the ones induced by WT-RELN. The polymicrogyria-associated variants Y1821H, G1280E, and R913C were the only variants generating rosettes with a cell body–poor center, 33%, 46%, and 29% of the time, respectively (Figure 4, Table 2, and Supplemental Figure 4). Notably, these variants also exhibited a higher propensity for inducing neuronal aggregation compared with WT-RELN. This was evidenced not only by the rostral aggregation in over 50% of electroporated brains (Figure 3C), not observed with the WT protein, but also by the strong increase in the average number of aggregates found per brain (Table 2). However, regarding the genetic context of patient C1, we observed that the de novo Y1821H variant seemed to have a more pronounced effect on RELN function compared with the G1280E variant. It induced a higher number of aggregates per brain (16.1 vs. 12.0), but with a lower proportion of rosettes (33% vs. 46%) (Table 2). Conversely, I650S and D556V variants identified in pachygyria patient DN* drove the formation of cell structures in which the GFP^+^ cells were spread throughout with their processes clearly misoriented, and, although expressing RELN, the mutant protein failed to accumulate in a central region (Figure 4 and Supplemental Figure 4). This resulted in structures completely lacking organization and cell body–sparse centers, which we defined simply as aggregates. Moreover, these variants displayed a lower capacity to induce neuronal aggregates, as indicated by the reduced number of aggregates per brain (Table 2), and did not form aggregates rostrally. Finally, cells electroporated with the C539R and R3207C variants expressed RELN, but they were unable to cause any sort of aggregate, and some GFP^+^ cells appeared arrested in the ventricular zone (VZ) (Figure 4, Table 2, and Supplemental Figure 4). Some of these neurons exhibited abnormal high levels of RELN intracellularly (Figure 4, white arrows), confirming the impairment of secretion detected in vitro (Figure 2B). All cell aggregates, whether affected or not, primarily consisted of later-born neurons expressing BRN2 found in superficial layers (Supplemental Figures 3 and 4), in accordance with the stage of electroporation and consistent with prior reports (54).

We conclude that all polymicrogyria-associated variants (Y1821H, G1280E, and R913C) can normally induce well-organized rosettes and are more prone to cause neuronal aggregation both caudally and rostrally. In contrast, variants associated with pachygyria (I650S, D556V, C539R, and R3207C) behave as LOF by altering the formation of rosettes or even aggregates to different extents, ranging from structures lacking organization and cell body–poor centers (I650S and D556V in DN*) to the complete absence of neuronal aggregation (variants in patients DN1 and DN2).

### RELN variants alter neuronal migration rostrally.

**Figure 5:**
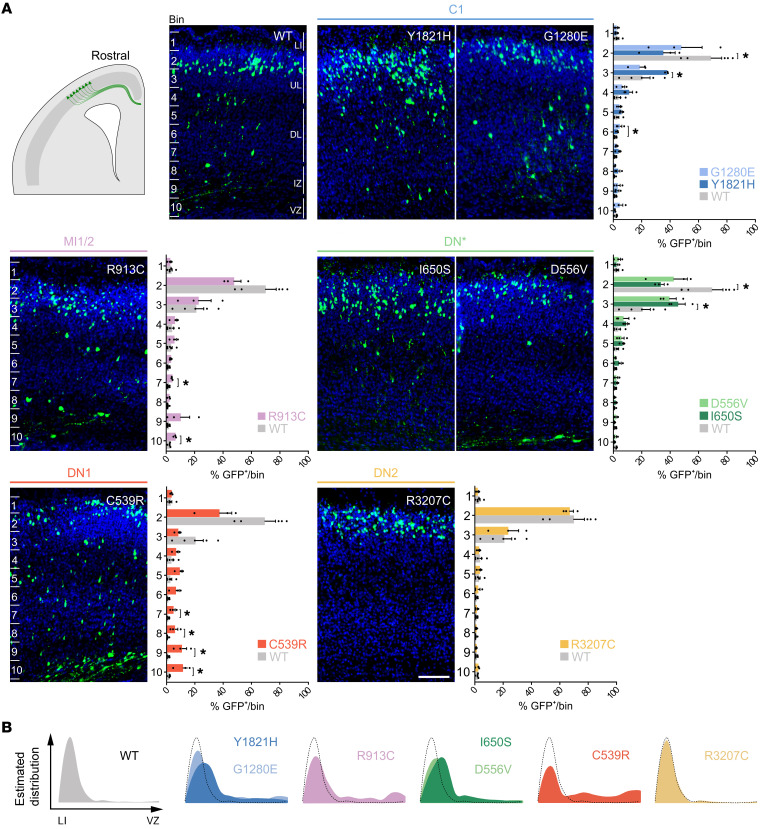
*RELN* variants affect cell migration at rostral levels. (**A**) Immunofluorescence wide-field images of P1 brains at rostral levels after IUE at E14.5. The entire thickness of the electroporated cortex was divided into 10 bins, and the percentage of electroporated GFP^+^ cells per bin was calculated (*n* = 5 WT-RELN, *n* = 3 mutants). Bin 1 corresponded to layer I (LI), bins 2–4 to upper layers (UL), bins 5–7 to deeper layers (DL), bins 8–9 to intermediate zone (IZ), and bin 10 to ventricular zone (VZ). Data are mean ± SEM; each symbol represents one electroporated brain; Kolmogorov-Smirnov test, *P < 0.05. Scale bar: 100 μm. (**B**) Recapitulative representation of the estimated distribution of electroporated cells from LI to the VZ for all constructs.

At rostral levels, where rosettes are not normally forming, WT-RELN–expressing GFP^+^ cells migrated to colonize the upper layers (UL) by P1, in particular layers II/III (LII/III), accordingly to the stage of electroporation (E14.5) (Figure 5A). Radially migrating neurons do not naturally express RELN; thus we used these electroporated principal neurons at rostral levels as a heterologous system to investigate the specific cell-autonomous effects of the different *RELN* variants on their migration. We divided the cortical wall in 10 equal bins and quantified the percentage of GFP^+^ cells per bin. Bin 1 corresponded to the MZ/LI, bins 2–4 to the UL, bins 5–7 to the deeper layers (DL), bins 8–9 to the IZ, and bin 10 to the VZ. When WT-RELN was ectopically expressed, 90% of GFP^+^ pyramidal neurons were found within bins 2–3 (70% in bin 2 and 20% in bin 3), corresponding approximately to LII/III as expected by the stage of electroporation. The remaining 10% of GFP^+^ cells were spread in the other bins (Figure 5, A and B). When the Y1821H and I650S variants were tested, defects were observed in the migration of the electroporated cells within the UL, with significantly fewer cells in bin 2 and more in bin 3. In contrast, the G1280E and R913C variants promoted an increase in the percentage of GFP^+^ cells migrating specifically in bins 6 and 7/10, respectively, corresponding to DL and VZ, despite no significant decrease in the percentage of neurons able to reach the UL (Figure 5, A and B). These findings indicate that the G1280E and R913C variants had a milder impact compared with the Y1821H and I650S variants. The D556V and R3207C variants did not affect the migration of the electroporated cells, thus behaving like the WT-RELN in this assay. The most striking effect was observed for the C539R variant, which strongly affected electroporated GFP^+^ cells with only 50% of them reaching the UL (bins 2–3) and the remaining being detected in deep locations, in particular in bins 7–10 (Figure 5, A and B), corresponding to DL (layers V/VI), IZ, and VZ.

To study whether disturbed migration was accompanied by changes in morphological features or fate, we analyzed both the cells that were displaced in the CP and those able to reach the correct position in the UL. Mislocalized cells for all variants displayed a morphology of migrating neurons with a long apical process accumulating RELN (Supplemental Figure 5A). De novo D556V, C539R, and R3207C variants appeared to cause an increased accumulation of RELN inside the cytoplasm of GFP^+^ cells (Supplemental Figure 5A, white arrows) correlating with the in vitro observations (Figure 2B). Cells that were able to reach the UL for both WT-RELN and the different variants appeared to differentiate normally into pyramidal neurons having their dendrites in LI and accumulating RELN mainly in the primary apical dendrite (Supplemental Figure 5C). Both mislocalized GFP^+^ cells in the CP (Supplemental Figure 5B) and those arrived in the upper CP (Supplemental Figure 5D) maintained the identity of BRN2^+^ upper-layer neurons for every variant as for the WT-RELN, showing that, even when mispositioned, electroporated cells maintained the correct upper-layer fate.

We conclude that the majority of variants alter cell-autonomously the migration of electroporated cells at rostral levels, although to different degrees, with the de novo C539R variant of DN1 being the most severely impaired.

### Pachygyria-associated de novo heterozygous RELN variants behave as dominant-negative forms in vitro.

**Figure 6:**
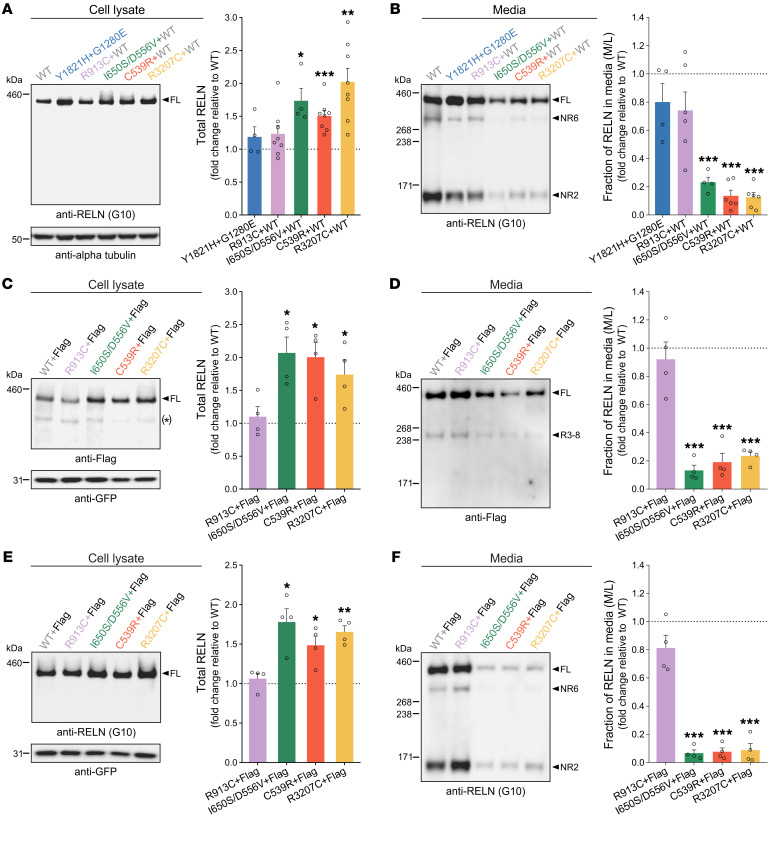
Pachygyria-associated de novo heterozygous *RELN* variants behave as dominant-negative in vitro. (**A** and **B**) Immunoblots (left) and densitometric analysis (right) of HEK293T cell lysates (**A**) and media (**B**) cotransfected with Y1821H and G1280E variants, or cotransfected with WT-RELN and R913C, I650S/D556V, C539R, or R3207C variants, probed with anti-RELN G10 or anti-GFP antibodies. RELN signal normalized to GFP in lysates and expressed as the media-to-lysate (M/L) ratio in the media (*n* = 4–8 independent transfections). (**C**–**F**) Immunoblots (left) and densitometric analysis (right) of cell lysates (**C** and **E**) and media (**D** and **F**) of HEK293T cells cotransfected with a FLAG-WT-RELN and WT-RELN, R913C, I650S/D556V, C539R, or R3207C variants, probed with anti-FLAG, anti-RELN G10, or anti-GFP antibodies. Data are presented as described for **A** and **B** (*n* = 4 independent transfections). All data are mean ± SEM; 2-tailed 1-sample *t* test, *P < 0.05, **P < 0.01, ***P < 0.001. Asterisk by immunoblot in **C** indicates unspecific bands. kDa, protein standard sizes.

To assess how RELN generated from mutant alleles might influence total RELN levels within the genetic context of the patients, we conducted in vitro cotransfection experiments using HEK293T cells. We replicated the heterozygous patients’ genotype by cotransfecting WT-RELN with either the R913C, I650S/D556V (carrying 2 variants in *cis*), C539R, or R3207C variants for patients MI1/2, DN*, DN1, and DN2, respectively. For C1’s two variants, we cotransfected Y1821H and G1280E to ascertain eventual combined effects on 2 different alleles. Western blot analysis of cotransfections mimicking C1 and MI1/2 genotypes showed unchanged amounts of RELN in both lysates and media (Figure 6, A and B), while DN*, DN1, or DN2 variants displayed at least a 1.5- to 2-fold increase in intracellular levels (Figure 6A) and a strong reduction of more than 70% of total secreted RELN (Figure 6B) compared with WT controls. These results indicate that Y1821H and G1280E mutant proteins, when both present, are secreted as efficiently as WT proteins. As for the monoallelic heterozygous variants, the coexistence of the MI1/2 variant form with the WT-RELN protein did not change secretion of RELN or its lysate levels, suggesting that this variant does not interfere with the WT protein. In contrast, the mutant carrying both I650S and D556V variants (I650S/D556V) and the C539R and R3207C variants seemed to strongly impair WT-RELN secretion while raising the amount of intracellular RELN (Figure 6, A and B), suggesting a dominant-negative effect. To further address whether these secretion-defective *RELN* variants have a dominant-negative effect on the WT protein, a C-t FLAG-tagged WT-RELN (20) (henceforth FLAG-WT-RELN) was cotransfected with either unflagged WT-RELN or RELN variants from the monoallelic heterozygous patients (MI1/2, DN*, DN1, and DN2). Western blotting with anti-FLAG antibodies showed that the I650S/D556V, C539R, and R3207C variants promoted an 80% decrease in secretion of FLAG-WT-RELN (Figure 6D), consistent with a 2-fold accumulation of intracellular RELN (Figure 6C). The FLAG-WT-RELN was identically secreted when coexpressed with either the WT-RELN or the R913C variant (Figure 6D). Similar results were obtained when total RELN was detected using N-t anti-RELN G10 antibodies (Figure 6, E and F). Altogether, these data demonstrate that the pachygyria-related variants generate secretion-defective RELN proteins that additionally act as effective dominant-negative in vitro.

To go further into the molecular mechanisms, we performed blots in non-reducing conditions to identify dimerized forms of RELN in coexpression experiments with FLAG-WT-RELN and monoallelic variants. As expected (12), a high proportion of RELN proteins in the media were present as dimers of around 900 kDa (Supplemental Figure 6A), and all monoallelic variants were capable of forming dimers with WT-RELN extracellularly. Additionally, in all conditions, we observed the presence of what seemed to be RELN multimers in the cellular fraction unable to enter the SDS-PAGE gel (Supplemental Figure 6B). This indicates that RELN assembles into large protein complexes also intracellularly, suggesting a possible mechanism through which the pachygyria-associated variants I650S/D556V, C539R, and R3207C retained the WT protein intracellularly (Figure 6C) and hindered its secretion (Figure 6D).

### Pachygyria-associated de novo monoallelic RELN variants behave as dominant-negative in vivo in both animal models and patients.

**Figure 7:**
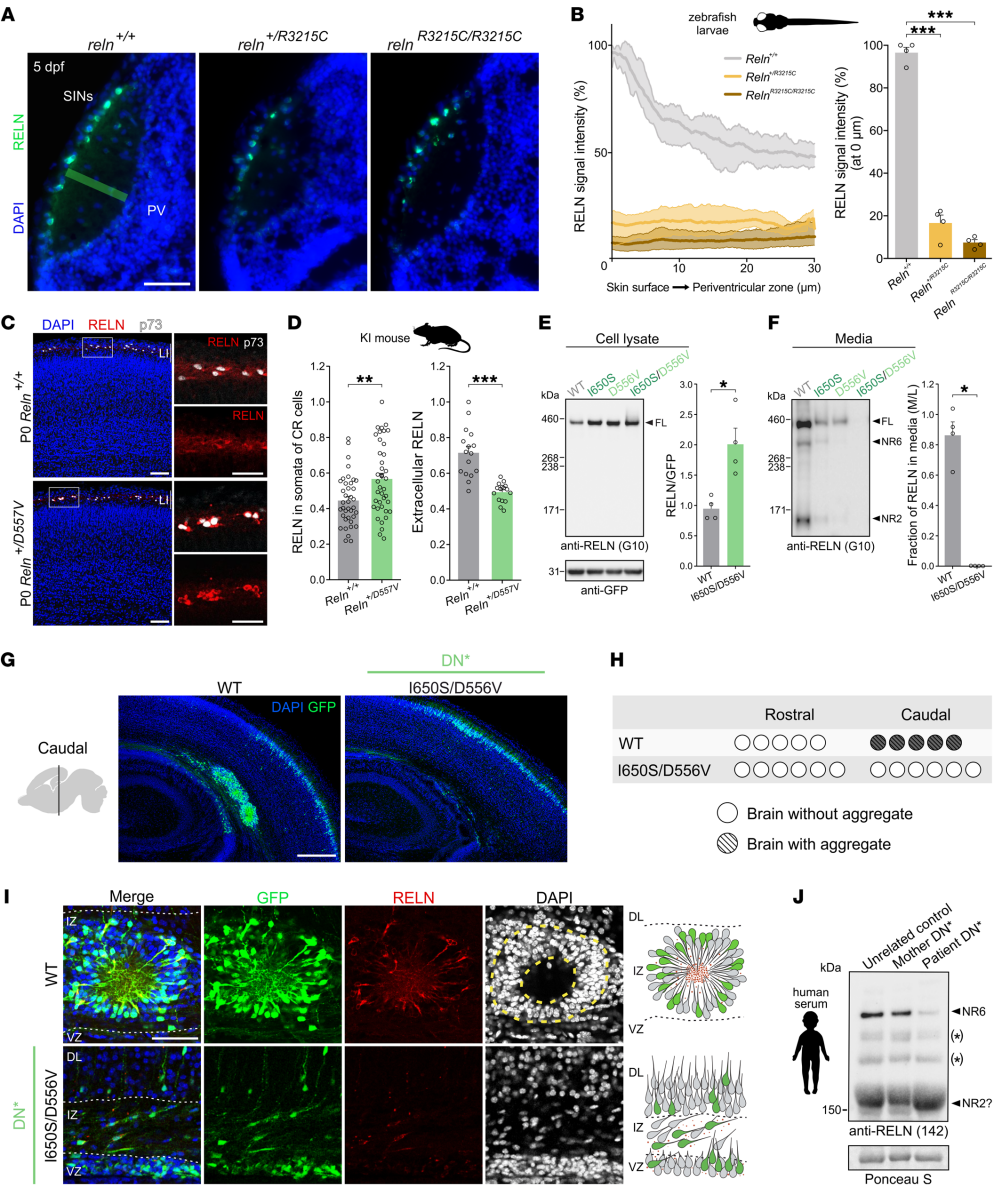
Pachygyria-associated de novo variants dominantly suppress RELN secretion in animal models and patients. (**A**) RELN (green) distribution in *Reln^+/+^*, *Reln^+/R3215C^*, and *Reln^R3215C/R3215C^* zebrafish at 5 days post-fertilization (dpf) on cryosectioned tecta, with DAPI (blue). PV, periventricular zone; SINs, superficial interneurons. Scale bar: 30 μm. (**B**) Densitometric plots (left) depict average RELN intensities (with minimum and maximum values) from the skin surface to the periventricular zone (green area in **A**) at distances 0, 10, 20, and 30 μm. Right: Fluorescence intensities at the neuropil surface. Data are mean ± SEM (*n* = 4 animals per genotype); 1-way ANOVA, Dunnett’s test, ***P < 0.001. (**C**) Immunofluorescence images of P0 *Reln^+/+^* and *Reln^+/D557V^* neocortices with CR cells expressing RELN (red) and p73 (white), with DAPI (blue). Scale bars: 75 μm. (**D**) RELN intensities in CR cell somata (*n* = 38 *Reln^+/+^*; *n* = 40 *Reln^+/D557V^* somata, from 4 brains per genotype) and in LI’s extracellular space (*n* = 16 regions of interest, from 4 brains per genotype). Data are mean ± SEM; Welch’s *t* test, **P < 0.01, ***P < 0.001. (**E** and **F**) Immunoblots (left) and densitometric analysis (right) of HEK293T lysates (**E**) and media (**F**) transfected with WT-RELN or RELN variants from patient DN*. RELN signal normalized to GFP in lysates and expressed as the media-to-lysate ratio in the media (*n* = 4 independent transfections). Data are mean ± SEM; Mann-Whitney test, *P < 0.05. (**G**) Immunofluorescence images of GFP^+^ (green) aggregates, with DAPI (blue), in caudal P1 mouse brains upon IUE at E14.5 of WT-RELN and I650S/D556V (*n* = 5–6). Scale bar: 250 μm. (**H**) Analysis of aggregate formation. (**I**) Immunofluorescence images of aggregates stained for GFP (green), RELN (red), and DAPI (blue). Scale bar: 50 μm. (**J**) Representative immunoblotting (from 2 experiments) of patient DN* blood serum, healthy mother, and unrelated control, with anti-RELN 142 antibodies. Ponceau S indicates equal protein loading. *Unspecific bands. kDa, protein standard sizes.

To assess the effect of *RELN* variants on its secretion in vivo, we turned to animal models and focused on the variants acting as dominant-negative in vitro. Previous reports revealed the gradient distribution of the RELN protein in the zebrafish optic tectum and its critical role for lamina-specific axonal targeting (55). Thus, we generated a zebrafish model recapitulating the genotype of patient DN2. Upon introduction of the R3215C point mutation, corresponding to human *RELN* R3207C in DN2, RELN spatial distribution was analyzed in embryos 5 days after fertilization (Figure 7, A and B). Anti-RELN immunostaining on tectal sections of R3215C WT sibling zebrafish embryos (*reln^+/+^*) revealed the local enrichment of RELN at the basement membrane and a gradual decrease toward the periventricular zone of the neuropil (Figure 7, A and B), similar to what was previously reported (55). In contrast, in *reln^+/R3215C^* and *reln^R3215C/R3215C^* mutants the RELN protein was detected in superficial interneurons, but a very weak extracellular localization or no clear extracellular localization of RELN could be detected in the heterozygotes and homozygotes, respectively, resulting in the abolishment of the gradient distribution of the protein (Figure 7, A and B). This suggests a strong reduction of its secretion within the neuropil due to the introduction of the R3215C mutation. More importantly, the substantial approximately 80% decrease of RELN in the heterozygous *reln^+/R3215C^* mutants aligns with the previous results obtained from the in vitro secretion assay (Figure 6, B, D, and F) and, thus, supports the dominant-negative effect of the human de novo R3207C variant also in vivo.

We were intrigued by the fact that, despite presenting pachygyria phenotypes, the single variants of patient DN* did not exhibit as severe defects in aggregate formation and secretion as did the 2 variants of patients DN1 and DN2. We thus decided to model in mice the de novo *RELN* D556V variant that was seen as the most defective based on the in vitro secretion and in vivo aggregation assays compared with the coexisting I650S variant. We generated heterozygous knockin (KI) mice carrying the point *Reln* mutation D557V, corresponding to the human D556V, and we found reduced extracellular RELN levels in LI of *Reln^+/D557V^* P0 cerebral cortices (Figure 7, C and D, right graph). Consistently, the amount of intracellular RELN was increased in the somata of p73^+^ CR cells (Figure 7, C and D, left graph), known to produce RELN in the developing neocortex (3, 4). This in vivo model thus reaffirms the human D556V variant as deleterious for RELN secretion in the cerebral neocortex. Given the co-occurrence of the de novo D556V variant with the inherited I650S variant in *cis* on the same allele, which produced a dominant-negative *RELN* variant in vitro (Figure 6), we sought to investigate whether the I650S/D556V variant worsened the effect on RELN secretion and function. Indeed, its amount was significantly raised in lysates of transfected HEK293T cells (Figure 7E) and totally absent in the culture media (Figure 7F) compared with WT-RELN, aggravating secretion defects with respect to each single variant alone (Supplemental Figure 7, A and B). We next assessed whether the I650S/D556V variant could prevent the capacity of RELN to form neuronal aggregates in vivo when ectopically expressed in the developing mouse neocortex. We showed that electroporated neurons with the I650S/D556V variant failed to cause neuronal aggregation in the IZ (Figure 7, G–I), contrary to either the WT-RELN or even its single variants I650S and D556V, like what was observed for the C539R and R3207C variants (Figures 3 and 4). These results demonstrate that RELN-dependent neuronal aggregation is abolished when both variants I650S and D556V coexist on the same protein, consistently with the strong dominant-negative behavior of the I650S/D556V variant observed in vitro (Figure 6).

Finally, to analyze RELN secretion in humans, we examined levels of blood serum RELN, which is mostly secreted from the liver (56), from patient DN*, the unaffected mother, and an unrelated control. The amount of the RELN fragment NR6, which is the most predominant form in human (Supplemental Figure 7C), rat, and mouse sera (56), was remarkably lower in the serum of patient DN* than in the sera from the healthy mother and control (Figure 7J and Supplemental Figure 7D). In samples prior to several freeze-thaw cycles, full-length RELN was also reduced in the affected child compared with the mother (Supplemental Figure 7D). The lower levels of serum RELN indicate impaired liver secretion of the altered proteins, and possibly reflect deficiency of secreted RELN in the brain of patient DN*. We thus conclude that the I650S/D556V variant is both a secretion-defective protein (Figure 7F) and a dominant-negative RELN form (Figure 6, B, D, and F) in vitro and in vivo.

Collectively, these results show that *RELN* missense variants alter different aspects of RELN secretion and function (Table 3). In particular, defects of in vitro/in vivo secretion and in vivo regulation of neuronal aggregation and/or migration align with the phenotypic features of the patients’ malformations, providing molecular insights into the cause of a broad spectrum of RELN-dependent NMDs. This functional characterization has contributed to improving the pathogenicity score of all variants, according to the ACMG 2015 guidelines, as proposed and summarized in Table 3.

## Discussion

*RELN* variants have been associated with a wide spectrum of neurodevelopmental disorders ranging from recessive forms of NMDs, namely LCH (57) with severe cerebral cortex and cerebellum malformations, to dominant ADLTE (44, 45), or psychiatric disorders such as autism and schizophrenia (41) with no apparent morphological brain abnormalities. Heterozygous *RELN* variants were also recently reported in individuals with mild LIS (37, 48), yet the underlying molecular mechanisms of such distinct pathological conditions remain unexplored. Here, we report 6 patients with heterozygous missense *RELN* variants, which expand the phenotypic spectrum of RELN-related cortical malformations to include pachygyria and polymicrogyria. Using complementary in vitro and in vivo assessments, we demonstrated that all heterozygous *RELN* missense variants linked to pachygyria severely prevented its secretion and neuronal aggregation activity, serving as causal to the disorder through a dominant-negative mechanism. Our findings also revealed that all tested polymicrogyria-associated variants maintained overall secreted RELN levels but presented an enhanced capacity to induce neuronal aggregation, suggesting their potential contribution to the pathology.

Among the 6 MCD patients, 3 patients (DN*, DN1, and DN2) share a similar phenotype consisting of frontal-predominant pachygyria, characterized by a simplified gyral pattern with broad gyri due to incomplete transmantle migration (51). Our functional studies demonstrated that all *RELN* variants associated with pachygyria (I650S/D556V, C539R, and R3207C) homogeneously behaved as LOF in the neuronal aggregation assay owing to the severe impairment of their secretion. Indeed, high levels of secreted and functional RELN are required to induce ectopic rosettes with cell body–sparse centers composed of leading processes and abundant extracellular RELN, similar to the MZ in vivo (54). These “mini-cortex”–like structures closely resemble the characteristic inside-out cell arrangement of the neocortex (54), which is determined by CR cells secreting RELN in the MZ to control radial neuronal migration (3). The secretion analysis in vitro and in vivo further revealed a dominant-negative effect on the WT protein, supporting a pathogenic role of I650S/D556V, C539R, and R3207C variants in causing a dominant form of pachygyria. The precise mechanism through which the mutant RELN proteins interfere with the WT protein remains undetermined. However, our immunoblotting results suggest that the presence of the mutant variants causes RELN retention in the intracellular compartment, possibly by “poisoning” the assembly of RELN multimers inside the cells. This assembly-mediated dominant-negative effect is frequently observed for proteins that form homomeric complexes (58), as reported for secreted RELN (12). This negative dominance can particularly explain the difference between patient DN* and the father, who carries the heterozygous I650S variant without any brain malformations. On one hand, the I650S variant alone, whose secretion was reduced to 50% in vitro, seems to be a benign variant concerning cortical malformations. Moreover, cortical layering in the heterozygous *Reln^+/D557V^* mouse (modeling the human de novo D556V) seemed typically normal despite the reduced levels of secreted RELN (70% secretion vs. control) in LI. This aligns with 50% of RELN being sufficient for a proper inside-out cortical lamination as observed in the heterozygous *reeler* mouse (30) and individuals with ADLTE (44, 46) or ASD (47) carrying heterozygous *RELN* variants. On the other hand, both variants I650S and D556V showed partial LOF in the in vivo aggregation assay, resembling to the disorganized RELN-induced aggregates previously described in a model with knockdown of Nrp1, a transmembrane protein that forms a complex with VLDLR to which RELN strongly binds (59). Taken together, these findings suggest that each individual variant could bind less efficiently to the Nrp1/VLDLR complex because of protein misfolding but, in isolation, is insufficient to develop cortical malformations. Notably, we showed that the occurrence of pachygyria in the child designated DN* is caused by the dominant-negative effect of the I650S/D556V variant resulting from the synergistic interaction of the 2 variants that severely hampers RELN secretion to 20% when both are present on the same allele as compared with their individual effects. This is indeed supported by the reduction of circulating RELN in the blood of patient DN*. Lastly, we believe this to be the first study replicating human *RELN* variants related to pathology in animal models. Both the KI *Reln^+/D557V^* mouse and *reln^+/R3215C^* zebrafish models showed in vivo alterations recapitulating the observed secretion deficiency of RELN in vitro, and more importantly, the mutant zebrafish, modeling the DN2 genotype (R3207C), revealed the damaging dominant-negative phenomenon of heterozygous variants in vivo, which severely drop RELN levels to 20%. Over the years, accumulating evidence has shown that RELN secreted by CR cells concentrates in the MZ and is necessary in various developmental events during cortical lamination, beginning with the initial preplate splitting (60), and extending through multiple steps of radial glia-independent neuronal migration. The latter include somal translocation for early-born neurons (27), followed by multipolar migration (61, 62) and terminal translocation for late-born neurons (63). Disruptions at any RELN-dependent steps can result in cortical malformations (64). Hence, all evidence supporting the pathogenicity of *RELN* variants identified in the pachygyria patients reflects deficient levels of functional RELN in their developing neocortex that potentially disrupt RELN-dependent neuronal migration from its early steps.

The other 3 patients (C1 and MI1/2) presented different forms of bilateral polymicrogyria. Polymicrogyria is an etiologically heterogeneous malformation characterized by overfolding and dyslamination of the neocortex thought to arise from late migration deficits and/or post-migrational abnormalities (64). In contrast to pachygyria patients, the polymicrogyria-related variants Y1821H, G1280E, and R913C revealed a GOF effect in our in vivo aggregation assay, firstly by promoting ectopic neuronal aggregation unusually in rostral brain regions, and secondly by increasing aggregation caudally. These functional alterations seem unrelated to their secretion levels, as both Y1821H and G1280E variants with normal secretion, and the variant R913C with lower secretion levels (60%), similarly enhanced aggregation in vivo. Furthermore, they successfully induced well-structured rosettes with a central cell body–free MZ-like region. In the developing neocortex, RELN signals to late-born migrating cortical neurons when they arrive beneath the MZ to organize the inside-out lamination (25) via an ApoER2-mediated mechanism regulating cell adhesion (61, 63, 65, 66), and to suppress neuronal invasion into the MZ via VLDLR (66, 67). These late developmental events are actually recapitulated in the RELN-induced aggregates, which involve both RELN receptors (54). This indicates that the binding to and signal transducing through ApoER2 and VLDLR receptors for all polymicrogyria-related variants should be intact. Nevertheless, all *RELN* variants displayed an enhanced rostro-caudal aggregation, shown to be facilitated by the direct promotion of N-cadherin–mediated cell adhesion of migrating neurons during both multipolar-bipolar transition and terminal translocation (65). This could suggest that the polymicrogyria-associated variants affect the adhesive properties of migrating neurons during possibly different steps of migration.

The R913C variant inherited from the mother, who has normal brain MRI but had epilepsy during childhood, was rare in gnomAD (~0.003%). Since whole-genome sequencing has yet to be performed for all polymicrogyria patients, the contribution of a deep intronic or regulatory variant in *trans* of the *RELN* variant or in another gene cannot be formally ruled out. However, NGS and whole-exome sequencing did not identify additional variants in the brothers MI1/2, which strongly reduces its probability. Concerning the Y1821H and G1280E variants in patient C1, the de novo occurrence of the Y1821H variant and its stronger associated effects in neuronal aggregation and migration in comparison with the G1280E variant allow the reclassification of Y1821H as pathogenic (Table 3) and suggest that it likely plays a critical role in the patient’s phenotype. Notably, the G1280E variant is proposed as a variant of uncertain significance (Table 3) despite its functional alterations, owing to its prevalence in around 1.2% of the normal population, with 11 reported homozygous individuals in the gnomAD (Non-neuro) database (68), indicating that alone it is not sufficient to develop a pathological condition. However, we cannot exclude the possibility that the combined effect of the de novo Y1821H and inherited G1280E variants may contribute to the phenotype, irrespective of the allelic configuration of both variants. Nevertheless, our data showed that the variants found in patients with polymicrogyria function differently from the WT, likely by promoting excessive neuronal adhesion, but are not dominant-negative, suggesting a contribution or predisposition to the manifestation of polymicrogyria in the patients rather than causality.

Many missense variants cause structural perturbations that may disrupt signal transduction through altered protein folding, protein-protein interactions, or receptor binding. In RELN, cysteine (Cys) residues are particularly important for both intramolecular disulfide bridges (69, 70) and homodimerization (13, 70), and 3 of the variants in this study involve cysteine-arginine interchanges. At the N-terminal region, which directs RELN non-covalent dimerization essential for its full biological activity, the C539R variant disrupts an intramolecular disulfide bond formed between the pair Cys^462^-Cys^539^ (69), thus impairing this bridge and RELN’s tertiary structure. Cys^462^ is left free to form new disulfide bonds and could interact with other Cys, including of the WT protein. The R913C and R3207C variants introduce new Cys in opposite domains of the protein, which could again affect correct folding and create new binding interfaces, such as intermolecular interactions with the WT protein. Nonetheless, the distinct phenotypes associated with R913C and R3207C variants pinpoint that an introduced Cys can actually generate opposite functional alterations, GOF or complete LOF, depending on protein domains, as demonstrated in this study. Regardless of their level of secretion, the specific structural alterations induced by the R913C and R3207C variants are crucial in exploring the significance of their intracellular and extracellular mechanisms of action to the associated pathology.

Our heterologous RELN expression assay in the developing mouse neocortex provided temporal and spatial control to assess the impact of all *RELN* variants on the radial migration of projection neurons without much interference from endogenous RELN. This assessment also allowed tracking of the migration of electroporated neurons from their birth in the VZ/subventricular zone to their final target position in the CP at rostral levels, in comparison with the WT counterpart. Our results showed that certain *RELN* variants led to the misplacement of electroporated neurons across specific layers of the cortical wall. These observations suggest that missense variants can alter RELN function in neuronal migration in a cell-autonomous manner, a mechanism yet to be assessed in vivo within the naturally RELN-expressing neurons, notably CR cells in the MZ and interneurons in the MZ, IZ, and CP. The multiple roles of RELN signaling in neuronal migration seem to rely on the distinct expression patterns (71) and function of its ApoER2 and VLDLR receptors (61, 66, 67). However, in the case of polymicrogyria-associated variants, the canonical RELN signaling cascade involving ApoER2/VLDLR appears to be unaffected based on the well-organized rosettes formed. Consequently, misplacement of electroporated neurons within the cortical layers suggests that alternative signaling pathways could be impacted, involving other RELN-binding transmembrane proteins that have been proposed to participate in the regulation of RELN-dependent neuronal migration steps and cortical layering (72). These include β_1_-containing integrins (23), and ephrin B/EphB tyrosine kinase receptors (73, 74), which are differentially distributed along the migratory route (75–77). Further investigation is necessary to explore the effect of these pathology-associated variants on non-canonical RELN signaling that could be responsible for the mispositioning of the migrating neurons. Notably, our data revealed that the dominant-negative C539R variant (DN1) significantly affected the migration of electroporated cells rostrally, but the R3207C (DN2) did not. This disparity in effect appears to be independent of secretion, as both variants presented similar impairments in secreted levels, suggesting that their distinct effects on migration mostly occur in the intracellular milieu upon their expression. However, in case a small amount of protein is still secreted, we must also consider disparities in protein signaling due to the conformational differences caused by each amino acid substitution. Altogether, our observations highlight possible, yet unknown, protein-protein interactions between RELN and the migration machinery, and a potential cell-autonomous role in the distribution of RELN-producing cells, for instance for CR cells, whose migration speed and consequent repartition in the developing neocortex are crucial for the patterning of higher-order cortical areas (78, 79).

None of the patients exhibited cerebellar anomalies previously shown as a hallmark of *RELN-*associated LCH (57). Like in the homozygous *reeler* mouse (4), the majority of previously reported NMD patients with *RELN* variants (32–34) exhibit a severely hypoplastic cerebellum that is associated with complete absence of RELN caused by protein truncation or a null allele. We describe here missense variants with a full-length protein generated but with negative dominance in vitro and in vivo, which do not affect the cerebellum, suggesting that RELN levels around 20% are sufficient for cerebellar but not cerebral cortex development. Notably, the heterozygous *reeler* mouse, which has 50% reduction of RELN protein, exhibits altered cortical circuits without disturbed layering and is considered a model for schizophrenia (30). Consistent with this observation, it was recently shown that half reduction of DAB1, an essential downstream signaling molecule in RELN signaling, reduces the neocortical LI thickness without defects in layer formation (80). Moreover, monoallelic missense *RELN* variants with hampered secretion in heterologous cells in vitro were associated with ADLTE (46) or ASD (47) without cortical malformations. Notably, ADLTE-linked variants impaired trafficking of mutant RELN toward the secretory pathway, leading to degradation instead (46), but the patients bearing the variants still showed significant levels of circulating RELN (44), which does not support a dominant-negative effect. The ASD-related *RELN* variants also exhibited decreased secretion without exerting a dominant-negative effect on WT-RELN secretion or affecting the downstream RELN signaling cascade (47). A common mechanism was suggested to underlie both RELN-dependent epilepsy and ASD, and is correlated with RELN haploinsufficiency, reducing protein levels to 50%. This possibly explains the insurgence of ADLTE or psychiatric disorders in the absence of cortical morphological abnormalities. Our work now shows that heterozygous *RELN* variants can lead to more severe phenotypes accompanied by altered cortical organization, as observed in our patient cohort. This is attributable to the dominant-negative effect of heterozygous *RELN* variants here observed that will reduce secreted RELN levels to 20% in individuals with pachygyria (DN*, DN1, and DN2). Finally, RELN secretion and thus overall WT-RELN and mutant RELN levels are not perturbed or mildly impaired (for the R913C variant) in polymicrogyria patients, suggesting that protein levels are not contributing to the pathology. Together, these results indicate that the occurrence of pachygyria or polymicrogyria, epileptic and psychiatric conditions, depends on the remaining distinct WT-RELN levels as well as on the role of specific variants in protein function.

In conclusion, we provide what we believe to be the first in vitro and in vivo functional characterization of *RELN* missense variants associated with NMDs. The outcomes of our functional studies allowed us to improve the pathogenicity scores for all tested variants, proposing that all patients may carry either pathogenic or likely pathogenic *RELN* variants (Table 3), and further highlighted the relevance of circulating RELN levels for diagnosis. This study paves the way for important functional assays for genotype-phenotype diagnostics to understand the mechanistic involvement of yet-to-be-identified *RELN* variants in NMDs. We correlate phenotypes of the described polymicrogyria and pachygyria patients with specific functional alterations of the RELN protein. Lastly, in addition to causing autosomal recessive NMDs (32), our results demonstrate that *RELN* variants also cause cortical malformations through dominant inheritance.

## Methods

Complete information on methods is provided in Supplemental Methods.

### Sex as a biological variable.

Sex was not considered as a biological variable. Both male and females animals were used in this study.

### Statistics.

Data are presented as mean ± SEM. Two-tailed 1-sample *t* test (hypothetical value of 1) after passing the Shapiro-Wilk normality test was used for statistical comparison of RELN levels obtained by Western blotting. For the migration assay, the non-parametric Kolmogorov-Smirnov test to compare cumulative distributions was performed. RELN intensity in the KI mouse was evaluated using unpaired parametric 2-tailed Welch’s *t* test, while the RELN gradient analysis in the larva tectum used 1-way ANOVA with Dunnett’s multiple comparisons as post hoc test. Analyses were performed using GraphPad Prism 7.0 software. *P* values less than 0.05 were considered significant (*P < 0.05, **P < 0.01, ***P < 0.001).

### Study approval.

Written and informed consent was obtained from all families prior to sample collection and processing. Animal procedures were performed in accordance with French and European Union animal welfare guidelines. In utero electroporation work was approved by the French Ministry of Higher Education, Research and Innovation as well as the Animal Experimentation Ethical Committee of Paris Descartes University (CEEA-34, license numbers 18011-2018012612027541 and 19319-2018020717269338), and zebrafish experiments were approved by the Charles Darwin Animal Experimentation Ethics Committee (CEEA 5) of Sorbonne Université (APAFIS#21323-2019062416186982). The mouse studies using the *Reln* D557V KI model were performed under the control of the Keio University Institutional Animal Care and Use Committee in accordance with the Institutional Guidelines on Animal Experimentation at Keio University.

### Data availability.

All data are included in the article and supplemental material, and values for all data points are provided in the Supporting Data Values file. NGS data of 2 patients were deposited in the Zenodo public database (https://zenodo.org/records/11381515). Additional information is available upon request.

## Author contributions

M Riva, SF, and AP conceptualized the study. RG, NBB, DJ, EP, EF, CB, and CJR performed clinical assessment. M Riva, SF, YS, Kotaro Hayashi, VPM, TH, Kanehiro Hayashi, CA, SA, M Rosello, JD, MS, OJH, and FC devised methodology. M Riva, SF, Kotaro Hayashi, Kanehiro Hayashi, KN, and AP performed investigation. M Riva, SF, NBB, and AP curated data. M Riva, SF, NBB, and AP wrote the original draft of the manuscript. M Riva, SF, YS, Kanehiro Hayashi, SA, FC, DJ, EP, RG, KN, FDB, NBB, and AP reviewed and edited the manuscript. M Riva, SF, Kotaro Hayashi, YS, SA, FC, NBB, and AP performed visualization. AP supervised the study and performed project administration. FDB, KN, and AP acquired funding.

## Supplementary Material

Supplemental data

Unedited blot and gel images

Supporting data values

## Figures and Tables

**Table 3:**
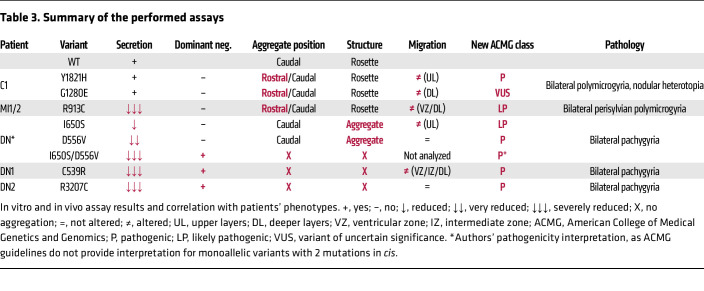
Summary of the performed assays

**Table 2:**
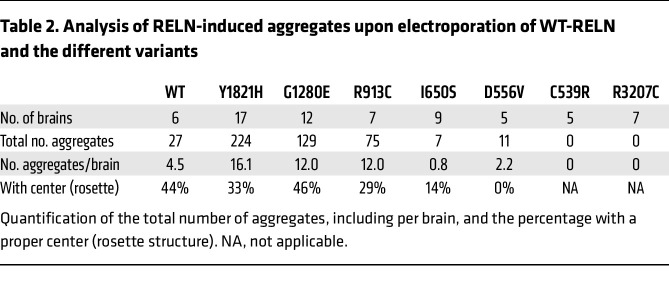
Analysis of RELN-induced aggregates upon electroporation of WT-RELN and the different variants

**Table 1:**
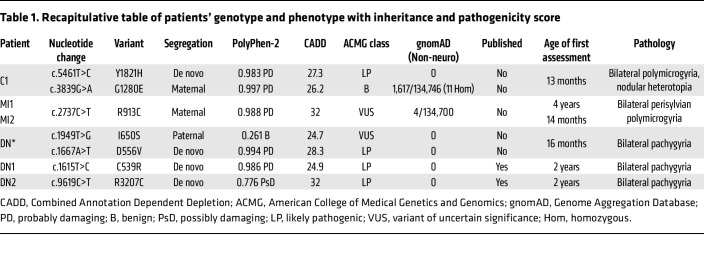
Recapitulative table of patients’ genotype and phenotype with inheritance and pathogenicity score

## Version 1 07/09/2024

In-Press Preview

## Version 2 08/15/2024

Electronic publication
