# Supplementary material for: De novo monoallelic Reelin missense variants cause dominant neuronal migration disorders via a dominant-negative mechanism
Source: J Clin Invest. 2024 Jul 9;134(16):e153097. doi: 10.1172/JCI153097 (PMC11324310; doi:10.1172/JCI153097)
Supplement: Supplemental data [file jci-134-153097-s271.pdf]

Supplemental Figure 1

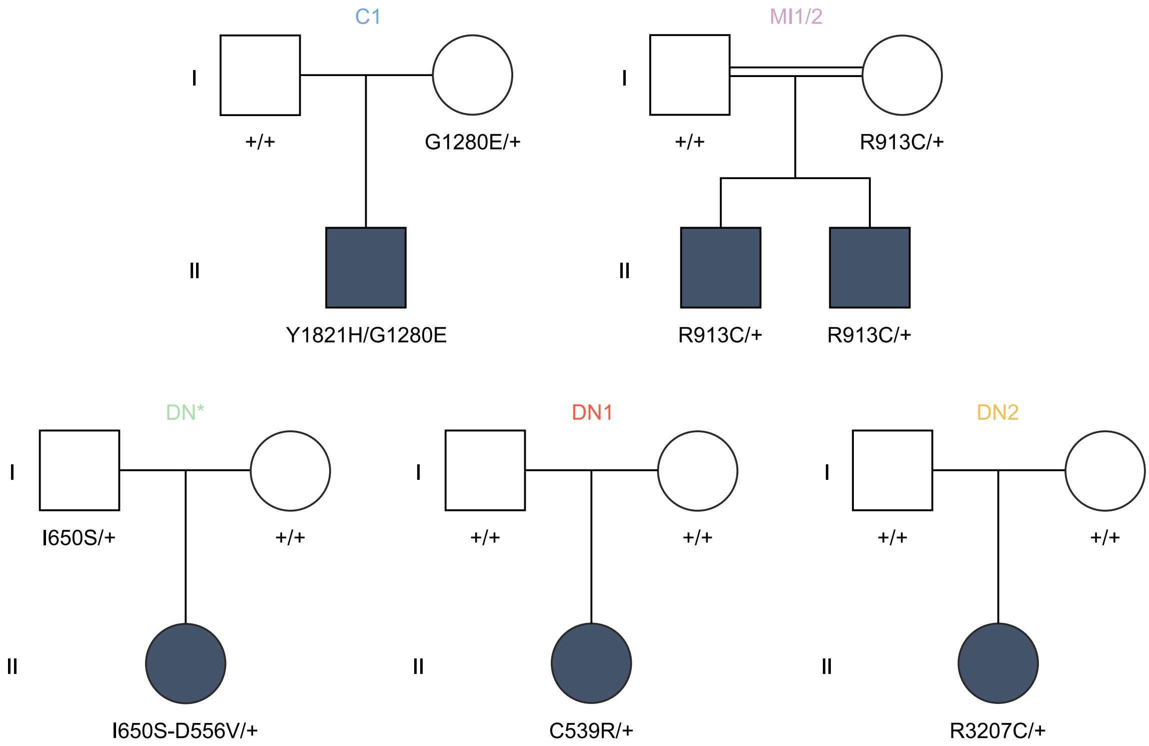

Supplemental Figure 1. Pedigrees of families showing segregation of RELN variants. Two generation (I, II) family trees of 5 families featuring NMDs are shown. Open and solid symbols indicate healthy family members and affected individuals, respectively. Squares indicate males and circles indicate females. Individuals with a RELN mutation (m) are indicated by m/m, m/+ and m-m/+. Individuals tested for mutations and found to be negative are indicated by +/+. Consanguinity is represented by a double horizontal line.

Supplemental Figure 2

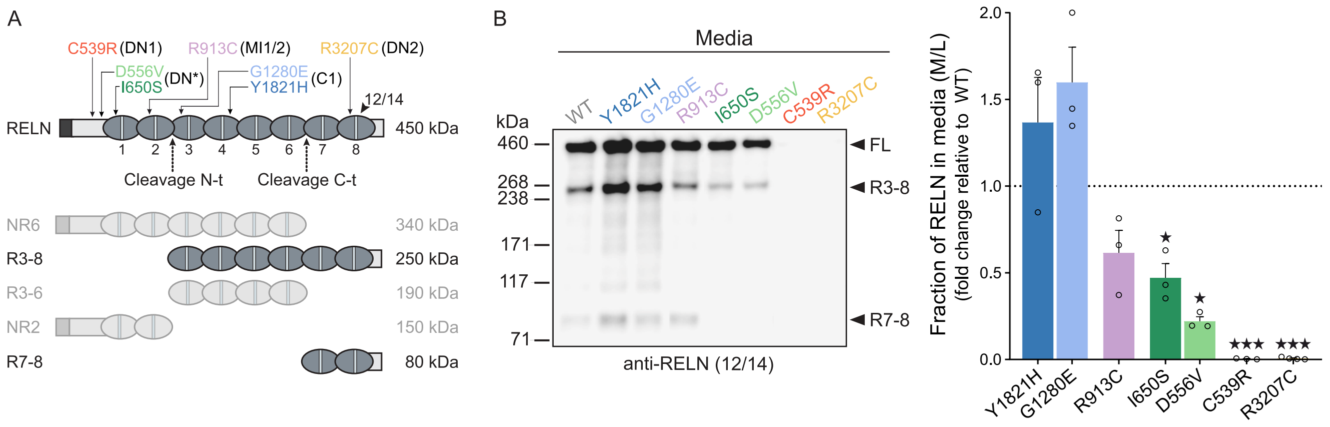

Supplemental Figure 2. Missense variants decrease RELN secretion in vitro using C-terminal 12/14 antibodies. (A) Schematic of the full-length (FL) RELN protein (450 kDa), its N-t and C-t cleavage sites (dashed arrows), and its five cleaved products (NR6, R3-8, R3-6, NR2, R7-8). The binding region of the 12/14 antibodies and the position of RELN variants are indicated with a black arrowheads and arrows, respectively. **(B)** Immunoblotting (left) of HEK293T cells media transfected with either WT-RELN or RELN-variants, probed with anti-RELN 12/14 antibodies. Densitometric analysis (right) of RELN signal is presented as the media(M)-to-lysate (L) ratio (n=3-4 independent transfections). Data are mean ± SEM; 2-tailed one sample t test, ★p<0.05, ★★★p<0.001. kDa, protein standard sizes.

Supplemental Figure 3

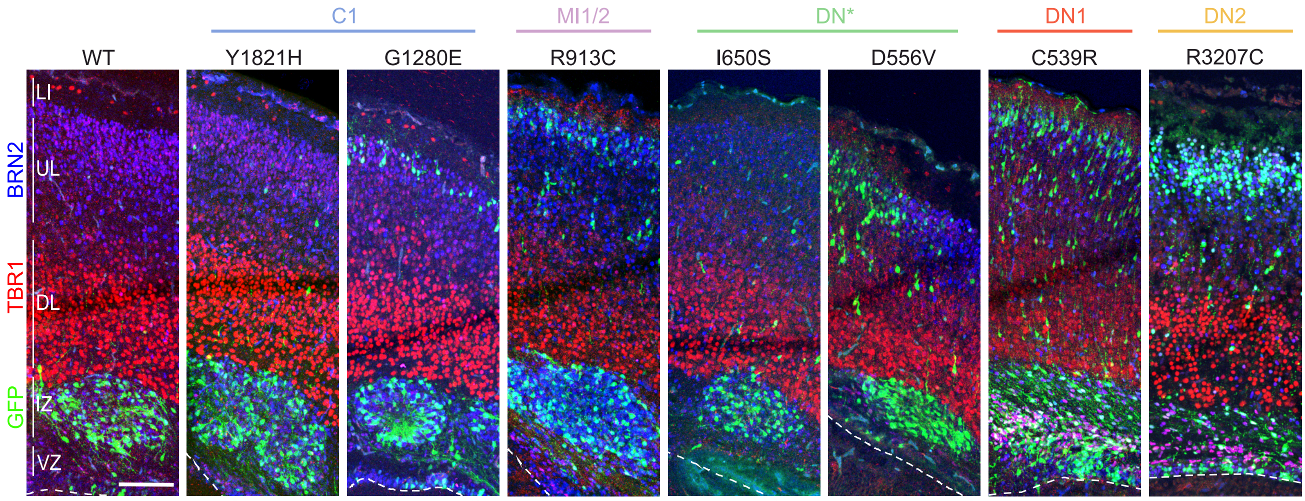

Supplemental Figure 3. Aggregates are located in the intermediate zone. Mosaic maximum projection confocal images showing the formed GFP$^+$ (green) aggregates localized in the IZ, immediately below TBR1$^+$ (red) neurons in deeper layers although expressing BRN2 (blue), a marker of upper layers. For the two variants C539R and R3207C, which fail to generate aggregates, numerous electroporated cells (green) are arrested in the VZ. Dashed lines delimit the ventricule. Scale bar, 100 µm. LI, layer I; UL, upper layers; DL, deeper layers; IZ, intermediate zone; VZ, ventricular zone.

Supplemental Figure 4

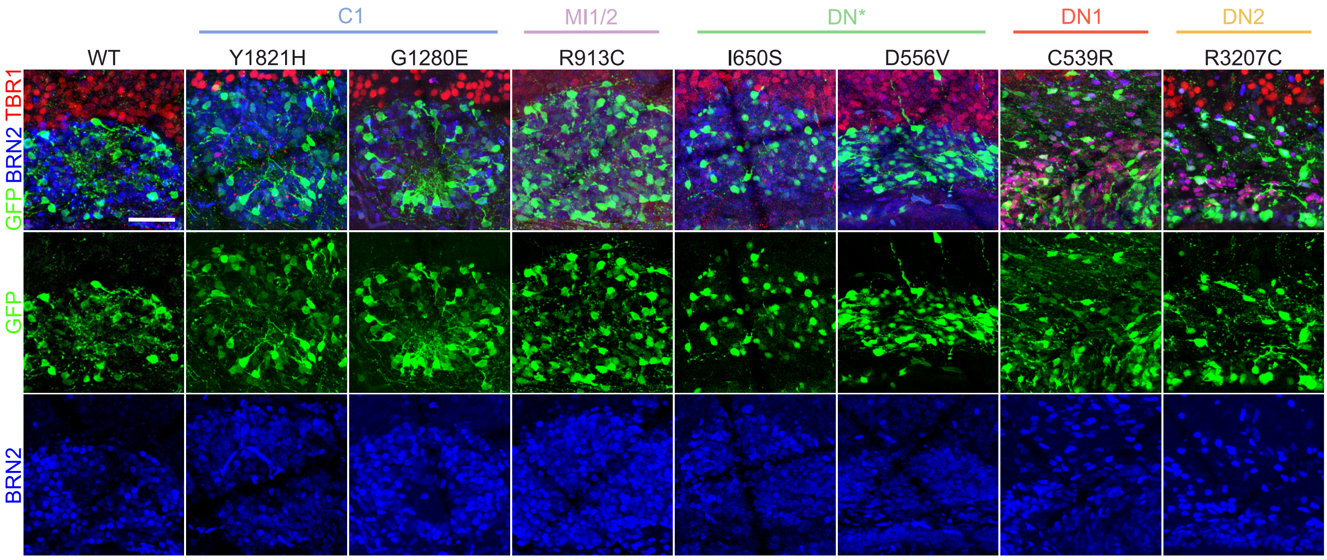

Supplemental Figure 4. Aggregates are composed by upper-layer neurons. Mosaic maximum projection confocal images of GFP^{+} (green) aggregates. According to the normal birthdating, neurons forming the aggregates located in the IZ below the $TBR1^{+}$ deeper layers (red) have an upper-layer neuronal fate expressing BRN2 (blue). For variants C539R and R3207C, cells arrested in the VZ are also $BRN2^{+}$. Discontinuities are due to mosaic stitching algorithm. Scale bar, 50 µm. Note: images in this figure are shown again in Supplemental Figure 3.

Supplemental Figure 5

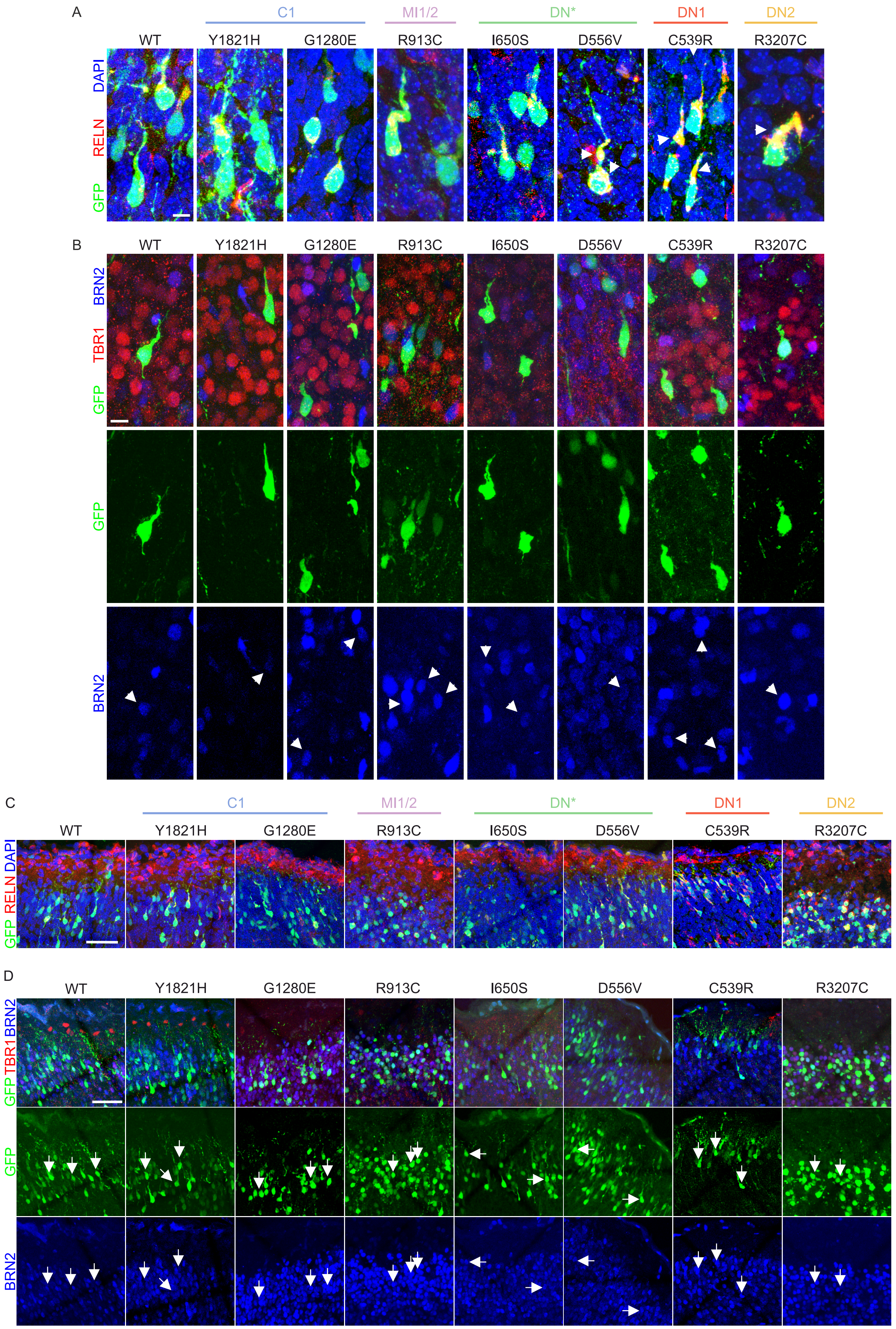

Supplemental Figure 5. Both electroporated neurons still migrating in the CP and those able to reach the upper CP differentiate correctly into upper-layer neurons. (A) Maximum projection confocal images of electroporated GFP$^+$ (green) neurons in the CP unable to reach the superficial layers at P1. Ectopic RELN (red) is accumulated in their apical processes. DAPI (blue) counterstaining. Cells electroporated with variants D556V, C539R and R3207C seem to accumulate higher RELN levels in their cytoplasm (white arrowheads). Scale bar, 5 µm. **(B)** GFP$^+$ pyramidal neurons (green) displaced in TBR1$^+$ deeper layers (red) still maintain upper-layer neuronal fate expressing BRN2 (blue, white arrows). Scale bar, 10 µm. **(C)** Mosaic maximum projection confocal images of electroporated GFP$^+$ (green) cells able to migrate and correctly differentiating into pyramidal neurons directing their dendrites into the RELN-enriched (red) layer I. DAPI (blue) counterstaining. Scale bar, 50 µm. **(D)** Mosaic maximum projection confocal images of electroporated GFP$^+$ (green) neurons reaching the top of the CP differentiate correctly into BRN2$^+$ upper-layer neurons (blue, white arrows). Scale bar, 50 µm.

Supplemental Figure 6

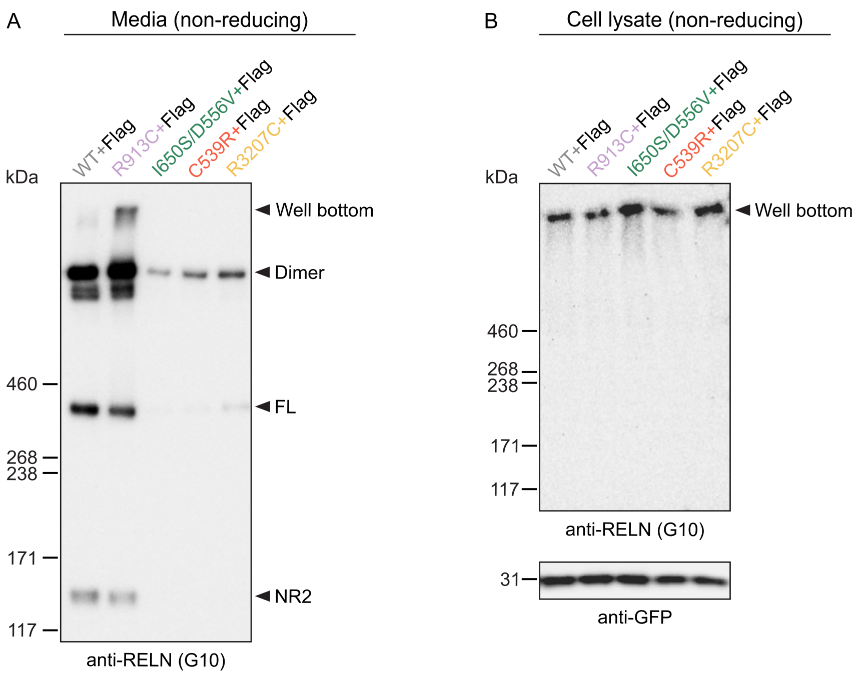

Supplemental Figure 6. RELN protein forms intracellular multimers. (A-B) Immunoblottings of non-reduced media (A) and non-reduced cell lysates **(B)** of HEK293T cells co-transfected with Flag-WT-RELN and WT-RELN, R913C, I650S/D556V, C539R or R3207C variants, probed with anti-RELN G10 or anti-GFP antibodies. Western blot samples were prepared without DTT and denatured by boiling (see Supplemental Methods). GFP detection was used as loading control as it migrated normally under non-reducing conditions. kDa, protein standard sizes.

Supplemental Figure 7

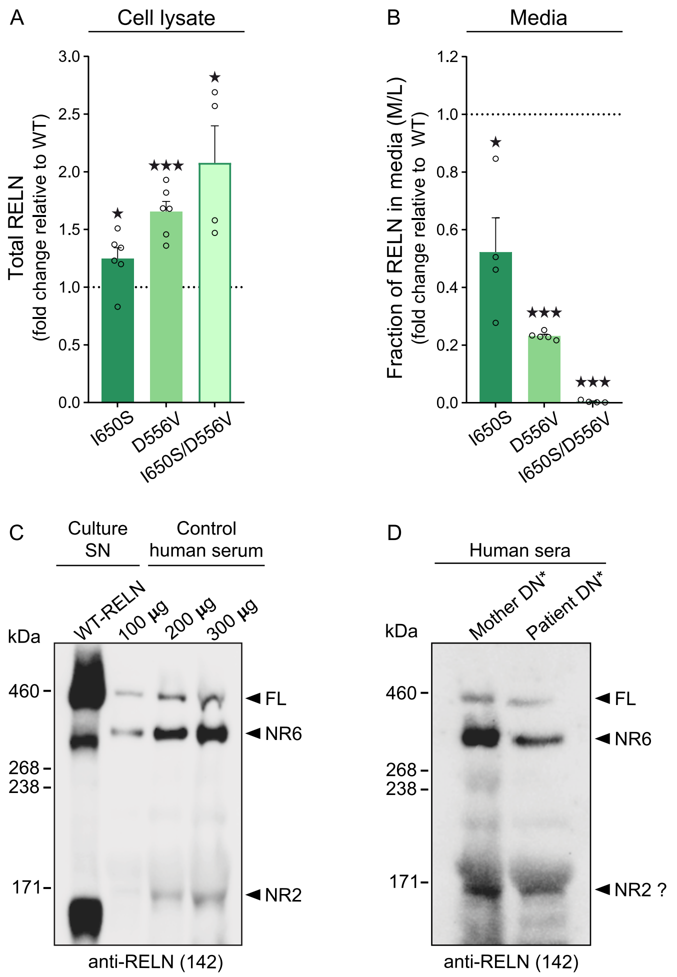

Supplemental Figure 7. DN*-belonging variants act in synergy to aggravate the deficit of RELN secretion. (A-B) Densitometric analysis of HEK293T cell lysates **(A)** and media **(B)** transfected with either WT-RELN or RELN-variants from DN* patient (alone or in cis), probed with anti-RELN G10 or anti-GFP antibodies. RELN signal normalized to GFP in lysates, and expressed as the media (M)-to-lysate (L) ratio in the media (n=4-6 independent transfections). Data are mean ± SEM; 2-tailed one sample t test, ★$p<0.05$, ★★★$p<0.001$. **(C-D)** Immunoblottings of blood serum probed with anti-RELN 142 antibodies: **(C)** from control subject with increasing protein quantities (µg) migrated along with recombinant WT-RELN from culture supernatant (SN); **(D)** from patient DN* and healthy mother. kDa, protein standard sizes.

SUPPLEMENTAL METHODS

Subjects

Six pediatric patients with radiologic evidence of abnormal cortical development were investigated. This selected cohort comprises patients who were ascertained directly by the authors: NBB (nadia.bahi-buisson@aphp.fr) and CB (celine.bellesme@aphp.fr) (C1 and DN*), DJ (dragana.josifova@gstt.nhs.uk) (MI1/2), RG (renzo.guerrini@meyer.it) and EP (elena.parrini@meyer.it) (DN1 and DN2), or whose clinical information and brain magnetic resonance images (MRIs) were sent to the authors. An expert neuroradiologist (CJR) trained in cortical malformations re-evaluated the MRIs. Venous blood (5-10 mL) from DN* patient, the mother and a control subject for western blotting was drawn into BD *Vacutainer® SST™* tubes and centrifuged at 1300 *g* for 10 min at RT. Plasma was collected and frozen at -80ºC until assay.

Genetic analysis

Genomic DNA for sequencing was extracted from peripheral blood samples. Genetic testing was performed in trios using a custom NGS panel targeting MCDs-associated genes (Supplemental Table 1).

For C1 and DN* patients, DNA libraries were sequenced on an Ion PGM™ sequencer (Thermo Fisher Scientific) following the manufacturer's instructions. The open-source Integrative Genomics Viewer (IGV) (81) and NextGENe® software (Next GENeration sequencing software for biologists) were used for data analysis. In silico analysis was performed using Polyphen, Polyphen2, SIFT, MutationTaster, and LRT tools to predict the pathogenicity of variants. For the DN* case, using the suppression of a restriction site (*AccI*) by DN*'s c.1667A>T mutation and a closed (612 bp) informative single nucleotide polymorphism (SNP), enzymatic digestion followed by PCR and Sanger sequencing showed the de novo D556V variant on the paternal allele.

For DN1 and DN2 patients, target enrichment and library preparation were performed using a custom-designed Nextera Rapid Capture assay (Illumina, San Diego, CA, USA). Sequencing was conducted on an Illumina MiSeq (Illumina, San Diego, CA, USA) with a 2x150 bp paired-end protocol as previously described (82). Variants were annotated and filtered using the ANNOVAR tool (83). Putative pathogenic variants were analyzed by Sanger sequencing to confirm the NGS results in probands and investigated in the parents to determine the inheritance status.

The consanguineous family of MI1/2 was also sequenced by WES using Agilent SureSelectXT Clinical Research Exome (SureSelectXT Human All Exon V5 baited with clinically relevant genes). The enriched exome libraries were sequenced using paired-end, 150-cycle chemistry on an Illumina NextSeq 550 sequencer (Illumina, Inc., San Diego, CA, USA) following the manufacturer's protocol. Data processing was carried out by mokapipe v2.0 (demultiplexing bcl2fastq, alignment BWA-MEM, variant calling, and coverage GATK). Cortical malformation and microcephaly virtual panels were applied. Horizontal coverage is determined using coding exons (+/- 5 bp) to a minimum depth of 20X. Assessment of pathogenicity was performed using QIAGEN Ingenuity Variant Analysis (CGI 54 Genomes, SIFT, EVS, Allele Frequency Community, JASPAR, Ingenuity Knowledge Base, Vista Enhancer, BSIFT, TCGA, PolyPhen-2, 1000 Genome Frequency, Clinvar, COSMIC, ExAC, HGMD, PhyloP, DbSNP, TargetScan), and Alamut for splice site analysis (SpliceSiteFinder-like, MaxEntScan, NNSplice, GeneSplicer). Validation was achieved by Sanger sequencing.
Variants classification followed the ACMG/AMP 2015 guidelines using the InterVar bioinformatic tool (84).

Mice

Pregnant SWISS mice used for in utero electroporation were purchased from Janvier Lab. The Reln D557V KI mouse was generated by using ICR mice (purchased from Japan SLC, Shizuoka, Japan) with CRISPR/Cas9. The target sequence of crRNA for the KI mouse generation was designed using CHOPCHOP (https://chopchop.cbu.uib.no/). Reelin D557 (GAC) was replaced into V (GTC) with five silent mutations to avoid repeated cleavage by Cas9 protein. Plugged ICR female mice after one day of mating were used for following *i*-GONAD procedure. The injection solution was prepared under the following conditions: 30 µM Alt-R® CRISPR-Cas9 crRNA (5'-TAATCGGAATGTCTGGGCTG-3', IDT, Coralville, IA, USA), 30 µM Alt-R® CRISPR-Cas9 tracrRNA (IDT), 2.0 µg/µl ssODN (5'-GACACCTGCCACCAAATTTTGTCTCAGGCAAAAGAGCCACCAAGGGTATAATCGC AACGTGTGGGCCGTAGTCTTCTTCCATGTGCTGCCCGTTCTCCCTTCAACAATGTC TCACATGATCCAGTTTTCT-3', Macrogen, Seoul, Korea), 1.0 µg/µl Alt-R® S.p. Cas9 Nuclease V3 (IDT, 1081058), 0.02% Fast Green in OPTI-MEM (11058-021, Thermo Fisher Scientific, Waltham, MA, USA). After this solution was incubated at RT for 5 min, *i*-GONAD procedure was performed as described previously (85) with slight modifications. The solution was injected into oviduct ampulla and electroporation was performed using a NEPA21 Super Electroporator (Nepa Gene, Chiba, Japan) as follows; poring pulse: 50 V, 5 msec pulse, 50 msec pulse interval, 3 pulse, 10% decay, pulse switch + and transfer pulse: 10 V, 50 msec pulse,

10 msec pulse interval, 3 pulse, 40% decay, pulse switch +/-. After birth, genomic DNA around the gRNA target region in each pup was amplified by PCR using primers 5’-CCTCACTGTCCACACAGAAATTC-3’ and 5’-CAGTTTACTGTGTATTGTCCTTCAGACA-3', cloned into pBluescript SK (-), and the sequence was confirmed by Sanger sequencing. For the genotyping of the KI mice, two sets of primers were used: 5’-AGGGTATAATCGGAATGTCTG-3’ and 5’-GGGATTCCTCTAAGACCTTTAC-3’ for the WT allele; 5’-AGGGTATAATCGCAACGTGTG-3’ and 5’-GTGGGATTCCTCTAAGACCTTTAC-3’ for the D557V allele.

Zebrafish mutant

Zebrafish (*Danio rerio*) were maintained as described previously (55) and housed in the animal facility of Institut de la Vision (Sorbonne University, Paris). Base-editing technology was used to generate the *RELN* R3215C mutation (corresponding to the R3207C in the human *RELN* gene) into the genome after C-to-T conversion. The pCS2+_*CBE4max-SpRY* plasmid (86) was linearized with *NotI* restriction enzyme and *CBE4max-SpRY* mRNAs were synthesized by an in vitro transcription with 1 µL of GTP from the kit added to the mix and a lithium chloride precipitation using the mMESSAGE mMACHINE sp6 Ultra kit (#AM1340, Ambion). Prior to injection, 2 µL of the crRNA (100 pmol/µL, 5’-AGTTCCGCTGGATCCAGATG-3’) and 2 µl of Alt-R® CRISPR-Cas9 tracrRNA (100 pmol/µL) from IDT were incubated at 95°C for 5 min, cooled down at room temperature (RT) and then kept on ice. To make the mutagenesis with base editing, a mix of 1 nL of *CBE4max-SpRY* mRNA (600 ng/µL) and the synthetic gRNA (43 pmol/µL) was injected into the cell at one-cell stage zebrafish embryos, which were raised until adult age. The fish carrying the mutation were identified by Sanger sequencing after PCR amplification of the locus using the following primers: 5’-CGGCCCTTTATTAATGATATCTCGTG-3’ forward primer and 5’-GTTTTAAAGAAGGAGCAGCAGAGTT-3’ reverse primer. After identification of the founders carrying the correct R3215C mutation by genotyping, they were then incrossed to produce the larvae analyzed in this study.

Plasmids

The full-length mouse *RELN* expression construct pCrl was kindly provided by Drs T. Curran and F. Tissir. The *RELN* cDNA was inserted using EcoRI and NotI restriction sites into a pCAG-IRES-NLS-EGFP vector containing a modified chicken β-actin promoter and a cytomegalovirus-immediate early enhancer (CAG) (87). Missense variants were inserted using

either the In-Fusion® HD Cloning Kit (Takara) following the manufacturer's protocols directly into the pCAG vector, or by PCR in pCrl, and then subcloned in the pCAG vector. Plasmid amplification used an EndoFree Plasmid Maxi Kit (Qiagen), with careful handling to avoid mechanical damage due to its large size (16 kb). The mouse protein shares 94.2% identity with the human protein at the aa level, with 3461 aa versus 3460 in human with an additional aa at the N-terminus. The human missense variants were conserved in the mouse sequence but at position +1aa and were cloned into the mouse RELN cDNA accordingly. The correct insertion of each point mutation was confirmed by enzymatic restriction and sequencing. The Flag-tagged WT-RELN constructed in a pCAGGS vector, a generous gift from Dr. M. Hattori, was described previously (20). The Flag-tagged WT-RELN construct contains a Flag-tag on the CTR and the single point mutation R3455A, which produces a WC (Within the CTR) cleavage-resistant RELN protein. This allows the detection by the anti-FLAG antibody upon secretion and it does not interfere with the formation of ectopic RELN-induced aggregates in the mouse brain.

Cell culture, transfection and sample preparation

Human embryonic kidney (HEK) 293T cells (ATCC CRL-3216) were cultured in Dulbecco's modified Eagle's medium (DMEM; Gibco) containing 10% fetal bovine serum (FBS) and 1% penicillin-streptomycin (Gibco) at 37ºC in a 5% CO_2 atmosphere. Cells were seeded onto 6-well plates at a density of 62500 cells/cm2 and cultured as previously described (18). Twenty-four hours later, transfection with 5 µg pCAG *RELN* constructs was performed at 80% confluence with Lipofectamine 2000 (Invitrogen) following the manufacturer's instructions. Mock-transfected cells (with transfection reagent only) and cells transfected with a pCAG-GFP plasmid served as controls. To phenocopy the heterozygous patients' genotypes and for the dominant negative assays, cells were co-transfected with 2.5 µg of each pCAG construct. To collect RELN-conditioned media, cells were maintained in serum-free Opti-MEM (Gibco) supplemented with antibiotics after 4 hrs of transfection and cultured for another 40 hrs. Conditioned media were collected, treated with a protease inhibitor cocktail (cOmplete™ tablets, Roche, Germany), cleared by centrifugation at 10000 rpm for 10 min and then concentrated at 4000 *g* for 30 min using Amicon Ultra 50K centrifugal filters (Millipore). Lysates were collected using RIPA buffer (50 mM Tris-HCl pH 7.5, 150 mM NaCl, 1% NP-40, 0.1% SDS, 0.5% sodium deoxycholate) supplemented with 2 mM EDTA and protease inhibitors (cOmplete™ EDTA-free tablets), then left in a rotator for 30 min at 4ºC to better dissolve the proteins followed by centrifugation at max speed for 10 min at 4ºC. Supernatants were stored at -20ºC until use. Protein quantification was determined using the bicinchoninic

acid (BCA) protein assay kit (Pierce™, ThermoFisher Scientific, USA) using bovine serum albumin (BSA) as standard.

Western blotting

Proteins samples were added to 1/4 volume of 4X LDS sample buffer (141 mM Tris, 106 mM Tris-HCl, 2% LDS, 10% glycerol, 0.51 mM EDTA, 0.22 mM SERVA Blue G, 0.175 mM phenol red, pH 8.5), to 1/10 of sample reducing agent 10X (50 mM DTT) and boiled at 95°C for 3 min. For non–reducing conditions no DTT was added. Protein samples (10 µg of cellular fraction; 2 µg of secreted fraction; 200 µg of human serum) were separated by SDS-PAGE on 3-8% tris-acetate gels (NuPAGE, Invitrogen) under reducing conditions at 150V (lysates and serum) or 110V (media) at RT and electro-transferred onto 0.45 µm nitrocellulose or PVDF membranes (Amersham, GE Healthcare, Germany) for 2h at 0.5 A at 4ºC. After 1 h blocking at RT with 5% (w/v) milk or BSA in Tris-buffered saline (50 mM Tris, 150 mM NaCl, pH 7.6) containing 0.1% Tween 20 (TBS-T), membranes were incubated overnight at 4°C with the following primary antibodies: mouse anti-RELN G10 (MAB5364, Millipore 1:1000), 142 (MAB5366, Millipore 1:400), mouse anti-RELN clones 12/14 (1:250, gift from Dr. André Goffinet), rabbit anti-GFP (A6455, Invitrogen 1:2000) and rabbit anti-alpha tubulin (PA529444, Invitrogen, 1:2000). After 3x10 min washes with TBS-T, the membranes were incubated with the appropriate HRP-conjugated secondary antibodies (Jackson Immunoresearch; 1:20000) diluted in TBS-T with 5% (w/v) milk for 1h at RT. After 3x10 min washes with TBS-T, blots were developed with SuperSignal West Pico Chemiluminescent Substrate or with SuperSignal West Femto Maximum Sensitivity Substrate (ThermoScientific) and visualized using the ChemiDoc apparatus (Bio-Rad). Quantitative analysis was determined by band densitometry using Image Lab™ software (Bio-Rad). The densities of protein bands were quantified with background subtraction. The RELN signal in lysates was normalized to GFP or alpha-tubulin, while RELN in the media (all three fragments) was determined as the ratio of RELN in the media to normalized RELN in lysates. The molecular weights were determined by using an appropriate pre-stained protein standard (HiMark 31-460 kDa, Invitrogen) for high molecular weight proteins.

In utero electroporation

The introduction of DNA vectors into cerebral cortical neurons was carried out by in utero electroporation as described previously (88), with some modifications. E14.5 timed-pregnant Swiss mice were deeply anesthetized with Isoflurane (AXIENCE SAS) at a concentration of 4% for induction and 2% for maintenance, and the uterine horns were exposed. A subcutaneous

injection of buprenorphine (0.05 µg/g) and ketoprophen (5 µg/g) was delivered as pre- and post-operation analgesia, respectively. Plasmids at a concentration of 4-7 µg/µl, containing Fast Green solution in a 1:10 ratio, were injected into one of the lateral ventricles with a glass micropipette. The embryo's head was carefully held between a pair of electrodes. Five electrical pulses of 40V and 50ms at intervals of 1s were delivered using a NEPA21 electroporator (Nepagene). The uterine horns were returned into the abdominal cavity to allow the embryos to continue normal development until birth.

Immunofluorescence

The birth date was considered P0. Electroporated P1 mouse pups were decapitated and the brains were dissected and fixed overnight by immersion in 4% paraformaldehyde (PFA) in 0.1M phosphate buffer (PB), pH 7.4 at 4°C. The next day brains were cryoprotected with 20% sucrose/PB overnight at 4°C. Brains were then embedded in OCT Compound (Sakura) and sectioned in 20 µm coronal slices along the rostro-caudal axis. P0 pups of *Reln* D557V/+ KI mice were perfused with ice-cold 4% PFA/PB, pH 7.4, and their brains were postfixed by immersion in 4% PFA for 2h at 4ºC. The brains were cryoprotected in 20% and 30% sucrose/PBS consecutively at 4ºC overnight. Then they were embedded in 75% OCT (OCT:30%sucrose = 3:1), frozen with liquid nitrogen and cryosectioned coronally at 20 or 30 µm thickness. Immunostainings on mouse was performed as previously described (79, 89, 90). Primary antibodies used were: mouse anti-RELN G10 (MAB5364, Millipore 1:1000), goat anti-RELN (AF3820, R&D, 1:300), chicken anti-GFP (GFP-1020, Aves Labs, 1:2000), goat anti-BRN2 (sc-6029, Santa Cruz biotechnology, 1:500), rabbit anti-TBR1 (ab31940, Abcam, 1:1000) and rabbit anti-p73 (ab40658, Abcam, 1:1000). Secondary antibodies used were: donkey Alexa-555 anti-mouse (A31570, Molecular Probes, 1:1000), donkey Alexa-488 anti-goat (A11055, Molecular Probes, 1:1000), donkey Alexa-488 anti-chicken (703-545-155, Jackson ImmunoResearch Laboratories, 1:1000), donkey Cy5 anti-goat (705-175-147, Jackson ImmunoResearch Laboratories, 1:500), donkey Cy3 anti-rabbit (711-165-152, Jackson ImmunoResearch Laboratories, 1:700) and donkey Alexa-555 anti-rabbit (A31572, Jackson ImmunoResearch Laboratories, 1:1000). Sections were counterstained with DAPI (5 µg/ml, D1306, ThermoFisher Scientific) and mounted in Vectashield (H-1000, Vector Labs). Zebrafish larvae at 5 dpf were fixed in 4% PFA, 1X PBS, pH 7.4, for 2h at RT or overnight at 4ºC. Mutant larvae were cryoprotected overnight in 30% sucrose/PBS, then embedded in OCT and sectioned at 14 µm thickness. Anti-RELN (MAB5366, Millipore, 1:200) immunostaining in tectal (zebrafish) cryosections was performed as described previously (55), for which antigen retrieval was performed using sodium citrate buffer.

Image acquisition and data treatment

Immunofluorescence images of electroporated mouse brains were acquired using a slide scanner Nanozoomer 2.0 (Hamamatsu) with a 20x objective or a Leica TSC SP8 inverted confocal microscope with 20x or 63x oil objectives (with or without digital zoom). Confocal images consist of multiple tile regions (mosaics) combined with serial z-stacks (0.5 µm through all the section's depth). Composite images are presented as maximum projections and were generated by the LAS X software using Mosaic Merge and Projection functions. Discontinuities are due to mosaic stitching algorithm. We assessed neuronal aggregation by examining brains with positive electroporated sites (GFP^+ cells) for the presence or absence of aggregates in rostral (pre-fimbria sections) and caudal regions (post-fimbria sections). Using fluorescent images captured by the Nanozoomer slide scanner, neuronal aggregates, including those with rosette structure, were manually counted in every brain section containing aggregates. For the migration assay in rostral regions, a portion of the electroporated section was divided in 10 bins and the number of GFP^+ neurons, detected by immunofluorescence, was counted using Adobe Photoshop CS4 software to determine the percentage of GFP^+ cells per bin.

For the analysis of *Reln* D557V/+ mice, immunofluorescence images were acquired with a Leica SP8 confocal microscope and a PL APO 20x/0.75 NA or a PL APO CS2 63x/1.40 NA oil-immersion objective lens (Leica Microsystems). With the 63x objective lens, each image was captured using z-stack series at intervals of 3.0 µm through all the section's depth. Using the Fiji software, the extracellular RELN intensity signal in regions of interest (ROIs; 5.05 µm x 5.05 µm) in the LI of the primary somatosensory cortex was measured (4 ROIs/brain, 4 brains for each genotype). Somata of Cajal-Retzius cells (CRs) was defined as ROIs based on DAPI, RELN and p73 stainings and RELN intensity signal were obtained (total 38 ROIs in WT and 40 ROIs in mutant, from 4 brains of each genotype).

Imaging of retinal cryosections from zebrafish was performed with a wide-field Zeiss M1 microscope 40x air objective. The RELN gradient analysis in zebrafish tecti was performed as previously described (55). The fluorescence intensity in the ROI (green rectangle in Figure 7A) was analysed with the Fiji software using the Plot Profile function and normalized to the average maximum fluorescence value of the ROI of the wild-type sample. Analysis was performed on neuropil tissue adjacent to the SINs, excluding cell bodies.

Supplemental Table 1: Gene lists included in the MCDs panel for NGS

Patient	MCDs Panel	Nº of genes
C1, DN*	***ACTB*** *(NM_001101.3)*, ***ACTG1*** *(NM_001199954.1)*, ***ARX*** *(NM_139058.2)*, ***CCND2*** *(NM_001759)*, ***CDC27*** *(NM_001114091.1)*, ***CUL4B*** *(NM_003588.3)*, ***DCX*** *(NM_178153.2)*, ***DYNC1H1*** *(NM_001376.4)*, ***ERMARD*** *(NM_018341.1)*, ***EML1*** *(NM_001008707.1)*, ***FLNA*** *(NM_001110556.1)*, ***GPR26*** *(NM_001145771.1)*, ***KIF1BP*** *(NM_015634.3)*, ***KIF2A*** *(NM_001098511.1)*, ***KIF5C*** *(NM_004522.2)*, ***MAP3K4*** *(NM_005922.2)*, ***NDE*** *(NM_001143979.1)*, ***NOTCH3*** *(NM_000435.2)*, ***OCLN*** *(NM_002538.3)*, ***PAFAH1B1*** *(NM_000430.3)*, ***RABGAP1*** *(NM_012197.3)*, ***RELN*** *(NM_005045.3)*, ***RTTN*** *(NM_173630.3)*, ***SNAP29*** *(NM_004782.3)*, ***TUBA1A*** *(NM_006009.2)*, ***TUBA8*** *(NM_018943)*, ***TUBB2B*** *(NM_178012.5)*, ***TUBB5*** *(NM_178014.2)*, ***TUBG1*** *(NM_001070.4)*, ***WDR62*** *(NM_001083961.1)*	30
MI1/2	Upon request to Dr. Dragana Josifova (Dragana.Josifova@gstt.nhs.uk)	
DN1, DN2	***ACTB*** *(NM_001101.3)*, ***ACTG1*** *(NM_001199954.1)*, ***ADGRG1*** *(NM_201525.4)*, ***AMPD2*** *(NM_001368809.2)*, ***ARFGEF2*** *(NM_006420.3)*, ***ARX*** *(NM_139058.2)*, ***ASNS*** *(NM_001673.5)*, ***ASPM*** *(NM_018136.5)*, ***ATRX*** *(NM_000489.6)*, ***CASK*** *(NM_001367721.1)*, ***CCND2*** *(NM_001759)*, ***CDK5*** *(NM_004935.4)*, ***CDK5RAP2*** *(NM_018249.6)*, *CENPJ (NM_018451.5)*, ***CEP135*** *(NM_025009.5)*, ***CEP152*** *(NM_001194998.2)*, ***COL4A1*** *(NM_001845.6)*, ***CUL4B*** *(NM_003588.3)*, ***DCHS1*** *(NM_003737.4)*, ***DCX*** *(NM_178153.2)*, ***DYNC1H1*** *(NM_001376.4)*, ***EMX2*** *(NM_004098.4)*, ***ERMARD*** *(NM_018341.1)*, ***EXOSC3*** *(NM_016042.4)*, ***EZH2*** *(NM_004456.5)*, ***FAT4*** *(NM_001291303.3)*, ***FIG4*** *(NM_014845.6)*, ***FKRP*** *(NM_024301.5)*, ***FKTN*** *NM_001079802.2)*, ***FLNA*** *NM_001110556.2)*, ***FOXC1*** *(NM_001453.3)*, ***GLI2*** *(NM_001374353.1)*, ***GNAQ*** *(NM_002072.5)*, ***IER3IP1*** *(NM_016097.5)*, ***ISPD*** *(NM_001101426.4)*, ***KATNB1*** *(NM_005886.3)*, ***KIF1BP*** *(NM_015634.3)*, ***KIF2A*** *(NM_001098511.1)*, ***KIF5C*** *(NM_004522.2)*, ***LAMA2*** *(NM_000426.4)*, ***LAMB1*** *(NM_002291.3)*, ***LAMC3*** *(NM_006059.4)*, ***LARGE1*** *(NM_133642.5)*, ***MCPH1*** *(NM_024596.5)*, ***NDE*** *(NM_001143979.1)*, ***NFIX*** *(NM_001365902.3)*, ***NHEJ1*** *(NM_024782.3)*, ***NSD1*** *(NM_022455.5)*, ***OCLN*** *(NM_002538.3)*, ***OPHN1*** *(NM_002547.3)*, ***PAFAH1B1*** *(NM_000430.3)*, ***PAX6*** *(NM_001368894.2)*, ***POMGNT1*** *(NM_017739.4)*, ***POMT1*** *(NM_001077365.2)*, ***POMT2*** *(NM_013382.7)*, ***PQBP1*** *(NM_001032382.2)*, ***QARS*** *(NM_005051.3)*, ***RAB18*** *(NM_021252.5)*, ***RAB3GAP1*** *(NM_012233.3)*, ***RAB3GAP2*** *(NM_012414.4)*, ***RARS2*** *(NM_020320.5)*, ***RELN*** *(NM_005045.3)*, ***RTTN*** *(NM_173630.3)*, ***SEPSECS*** *(NM_016955.4)*, ***SHH*** *(NM_000193.4)*, ***SIX3*** *(NM_005413.4)*, ***SLC25A19*** *(NM_001126121.2)*, ***STIL*** *(NM_001048166.1)*, ***TGIF1*** *(NM_003244.4)*, ***TMEM5*** *(NM_014254.3)*, ***TSC1*** *(NM_000368.5)*, ***TSC2*** *(NM_000548.5)*, ***TSEN15*** *(NM_052965.4)*, ***TSEN2*** *(NM_025265.4)*, ***TSEN34*** *(NM_001077446.4)*, ***TSEN54*** *(NM_207346.3)*, ***TUBA1A*** *(NM_006009.2)*, ***TUBA8*** *(NM_018943)*, ***TUBB*** *(NM_178014.4)*, ***TUBB2A*** *(NM_001069.3)*, ***TUBB2B*** *(NM_178012.5)*, ***TUBB3*** *(NM_006086.4)*, ***TUBG1*** *(NM_001070.4)*, ***VLDLR*** *(NM_003383.5)*, ***VRK1*** *(NM_003384.3)*, ***WDR62*** *(NM_001083961.1)*	86

SUPPLEMENTAL REFERENCES

81. Robinson JT, et al. igv.js: an embeddable JavaScript implementation of the Integrative Genomics Viewer (IGV). *Bioinformatics*. Jan 1 2023;39(1)doi:10.1093/bioinformatics/btac830

82. Parrini E, et al. Diagnostic Targeted Resequencing in 349 Patients with Drug-Resistant Pediatric Epilepsies Identifies Causative Mutations in 30 Different Genes. *Hum Mutat*. Feb 2017;38(2):216-225. doi:10.1002/humu.23149

83. Yang H, Wang K. Genomic variant annotation and prioritization with ANNOVAR and wANNOVAR. *Nature protocols*. Oct 2015;10(10):1556-66. doi:10.1038/nprot.2015.105

84. Li Q, Wang K. InterVar: Clinical Interpretation of Genetic Variants by the 2015 ACMG-AMP Guidelines. *American journal of human genetics*. Feb 2 2017;100(2):267-280. doi:10.1016/j.ajhg.2017.01.004

85. Ohtsuka M, et al. i-GONAD: a robust method for in situ germline genome engineering using CRISPR nucleases. *Genome Biol*. Feb 26 2018;19(1):25. doi:10.1186/s13059-018-1400-x

86. Rosello M, et al. Disease modeling by efficient genome editing using a near PAM-less base editor in vivo. *Nat Commun*. Jun 15 2022;13(1):3435. doi:10.1038/s41467-022-31172-z

87. Niwa H, et al. Efficient selection for high-expression transfectants with a novel eukaryotic vector. *Gene*. Dec 15 1991;108(2):193-9. doi:10.1016/0378-1119(91)90434-d

88. Tabata H, Nakajima K. Labeling embryonic mouse central nervous system cells by in utero electroporation. *Dev Growth Differ*. Aug 2008;50(6):507-11. doi:10.1111/j.1440-169X.2008.01043.x

89. Bielle F, et al. Multiple origins of Cajal-Retzius cells at the borders of the developing pallium. *Nat Neurosci*. Aug 2005;8(8):1002-12.

90. de Frutos CA, et al. Reallocation of Olfactory Cajal-Retzius Cells Shapes Neocortex Architecture. *Neuron*. Oct 19 2016;92(2):435-448. doi:10.1016/j.neuron.2016.09.020
