# Supplementary material for: De novo monoallelic Reelin missense variants cause dominant neuronal migration disorders via a dominant-negative mechanism
Source: J Clin Invest. 2024 Jul 9;134(16):e153097. doi: 10.1172/JCI153097 (PMC11324310; doi:10.1172/JCI153097)

Full unedited blots for Figure 2B

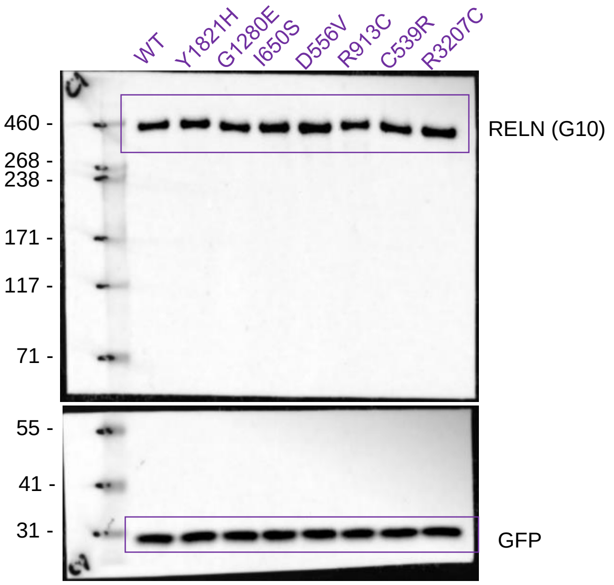

Full unedited blot for Figure 2C

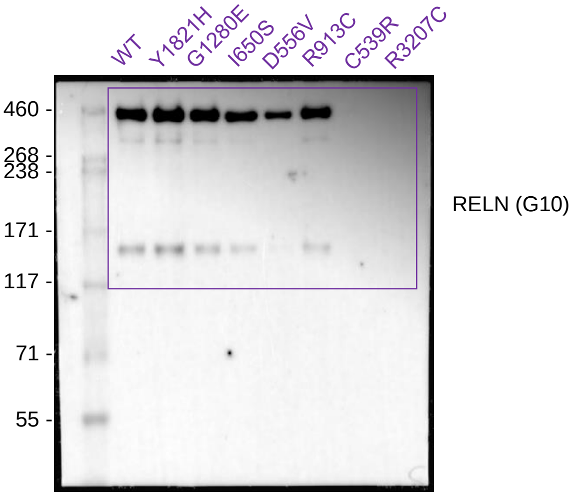

Full unedited blots for Figure 6A

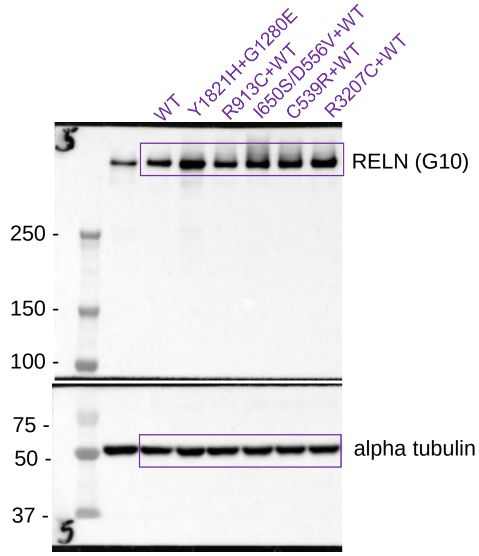

Full unedited blot for Figure 6B

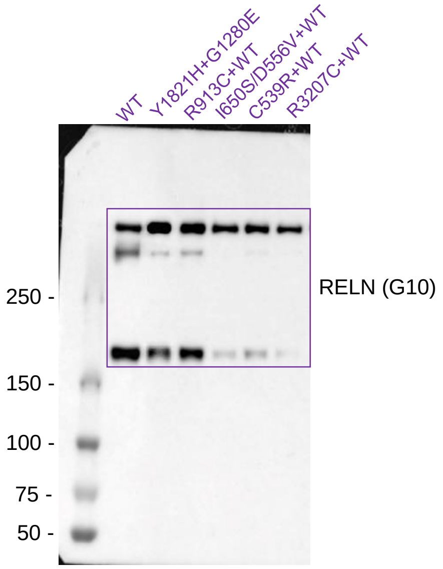

Full unedited blots for Figure 6C

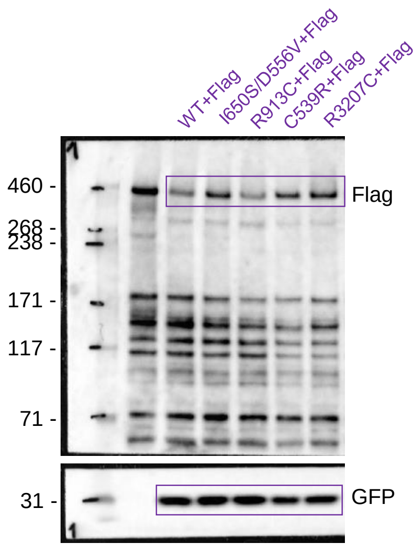

Full unedited blot for Figure 6D

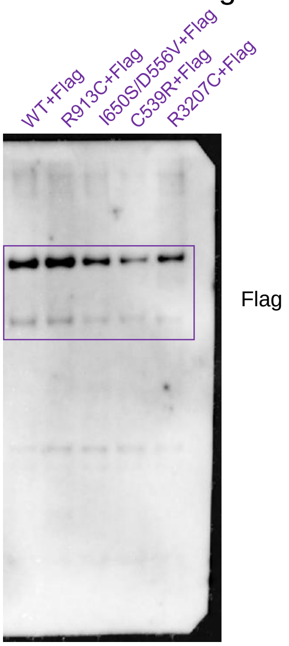

Full unedited blots for Figure 6E

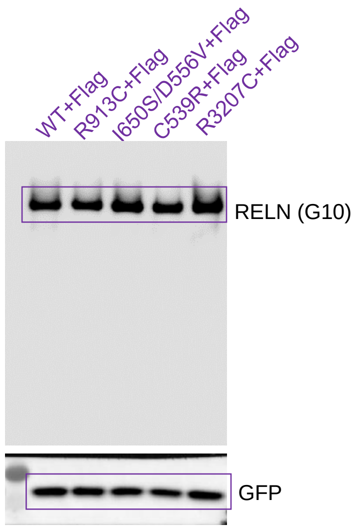

Full unedited blot for Figure 6F

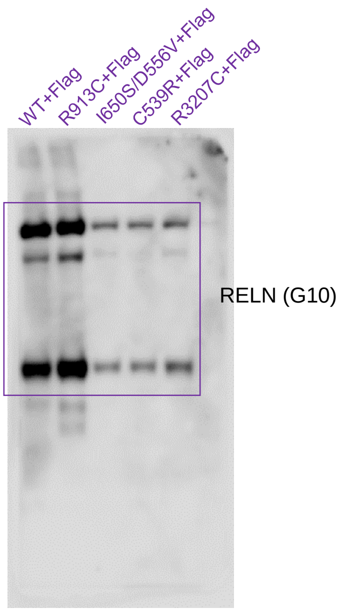

Full unedited blots for Figure 7E

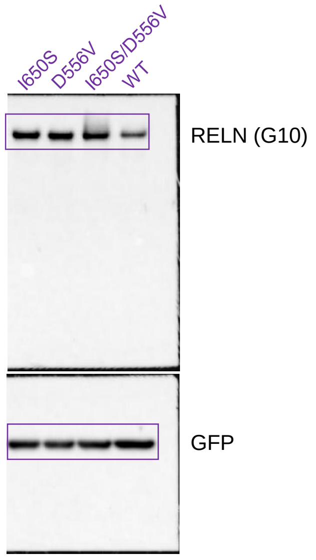

Full unedited blot for Figure 7F

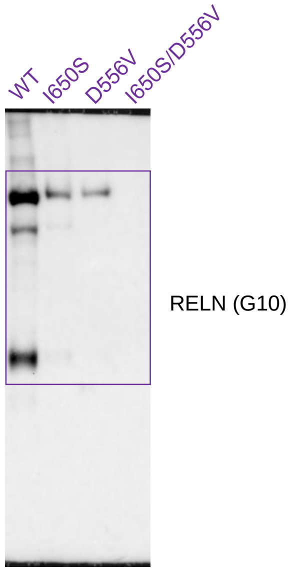

Full unedited blot for Figure 7J

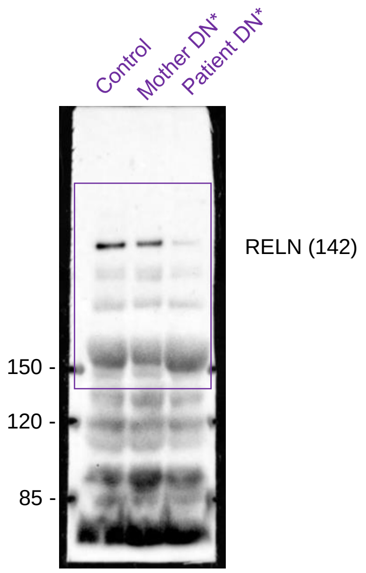

Full unedited blot for Supplemental Figure 2B

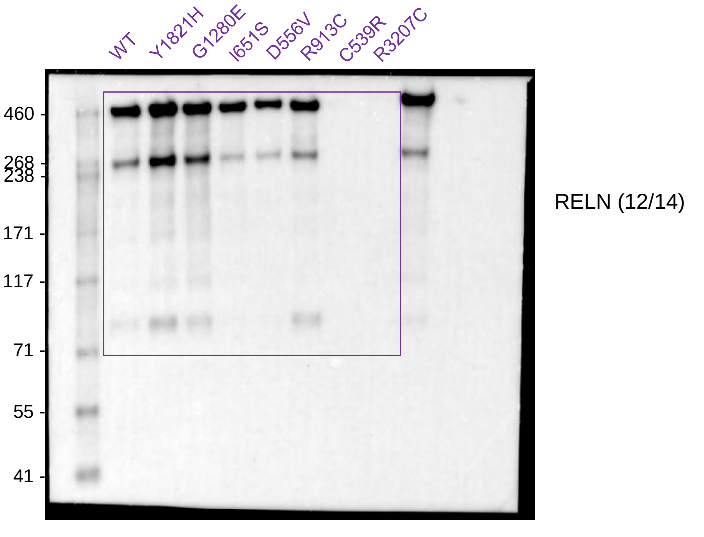

Full unedited blot for Supplemental Figure 6A

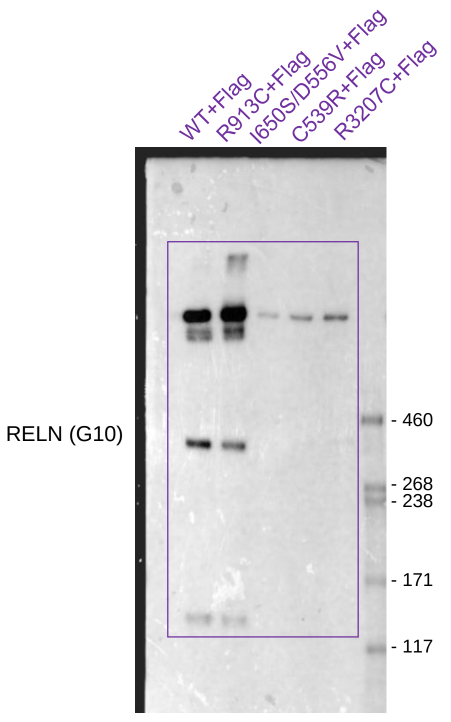

Full unedited blot for Supplemental Figure 6B

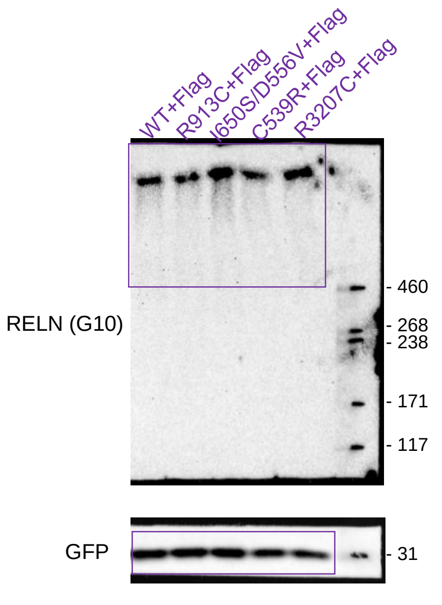

Full unedited blot for Supplemental Figure 7C

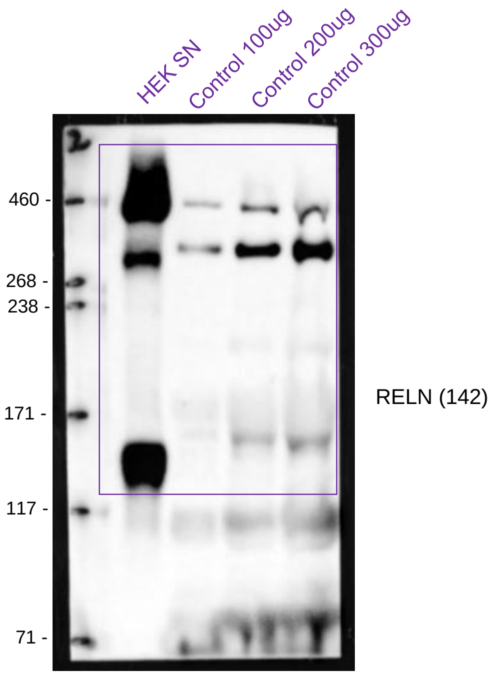

Full unedited blot for Supplemental Figure 7D

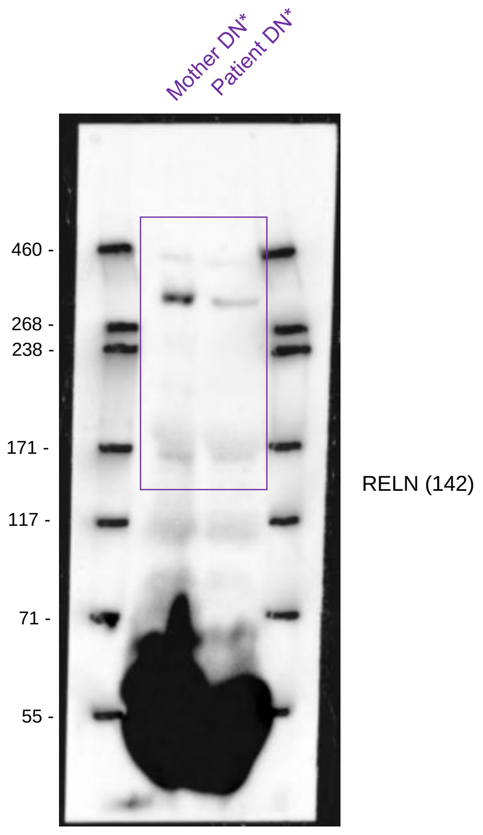

Supplement: Unedited blot and gel images [file jci-134-153097-s272.pdf]
